# Co‐Designing Lung Cancer Rehabilitation Services for People Treated With Immunotherapy

**DOI:** 10.1111/hex.70660

**Published:** 2026-04-05

**Authors:** Lara Edbrooke, Elizabeth Pearson, Catherine L. Granger, Linda Denehy

**Affiliations:** ^1^ Department of Physiotherapy The University of Melbourne Melbourne Australia; ^2^ Centre for Health Services Research in Cancer, Peter MacCallum Cancer Centre Melbourne Victoria Australia; ^3^ Sir Peter MacCallum Department of Oncology The University of Melbourne Melbourne Victoria Australia

**Keywords:** co‐design, immunotherapy, lung cancer, rehabilitation

## Abstract

**Background:**

Immunotherapy has rapidly become part of lung cancer care, however the ability of people to participate in rehabilitation in conjunction with immunotherapy remains largely unknown. The aims of this study were to (a) understand stakeholder perspectives of lung cancer immunotherapy treatment and (b) co‐design a rehabilitation programme for people with lung cancer during and following immunotherapy.

**Methods:**

Experience‐based co‐design, involving three distinct online phases: (1) patient, carer and healthcare professional interviews to understand immunotherapy and rehabilitation experiences; (2) separate workshops for healthcare professionals and patients/carers, to identify priority areas for future rehabilitation programme design; and (3) combined workshops to refine the draft programme. Transcripts were analysed by two researchers, guided by the Consolidated Framework for Implementation Research.

**Results:**

Seventeen stakeholders were involved in the interviews and/or workshops: seven patients, one carer and nine exercise healthcare professionals. Key themes included: immunotherapy side effects varied but were generally more tolerable than chemotherapy; rehabilitation information was lacking, and access varied; healthcare professional training in immunotherapy was limited. Rehabilitation enablers included supervision and monitoring from healthcare professionals with expertise in cancer; routine rehabilitation discussion with oncologists and nurses; dedicated funding. Lack of education about symptom control when exercising was a barrier for patients. Essential rehabilitation elements included individualised programmes, group‐based, flexibility for centre‐ or home‐based programmes. Programme components included education, aerobic and strength exercise and screening for nutrition and psychology needs.

**Conclusions:**

This research has furthered understanding of the lung cancer immunotherapy treatment journey and identified key design requirements of a rehabilitation programme.

**Patient or Public Contribution:**

Two patient advocates contributed to this study as members of the project steering committee. Their contributions for this study included reviewing and providing feedback on the proposed study design and the interview and workshop topic and question guides. Patient, carer and healthcare professionals co‐designed the rehabilitation programme through individual interviews and a series of small‐group workshops.

## 1 Background

People with non‐small cell lung cancer (NSCLC) experience high symptom burden and progressive functional decline, including reduced physical activity and muscle wasting [1, 2, 3, 4]. These impairments contribute to reduced health‐related quality of life (HRQoL) and mood disturbance. Traditional lung cancer treatments—including surgery, chemotherapy and radiotherapy—place a substantial burden on patients and affect multiple body systems [5]. When combined these disease and treatment‐related factors significantly impact behaviours related to well‐being: participation in activities of daily living, social, and family roles [6, 7] and the sequelae can persist for years [8]. Promisingly, new and more personalised treatment options, including immunotherapies, have rapidly become part of usual care for many people with NSCLC (referred to as ‘lung cancer’ hereafter).

Immunotherapy treatments activate the patient's immune system. They work to block proteins, known as immune‐checkpoint inhibitors (e.g., Programmed cell death protein 1 [PD‐1] and Programmed cell death ligand 1 [PD‐L1]) and enhance the ability of T‐cells to identify and destroy cancer cells [9]. Phase III trials support the use of immunotherapy monotherapies or chemoimmunotherapies in first‐line treatment of metastatic lung cancer in patients with good performance status [10]. Improvements in overall and progression‐free survival are seen in people with lung cancer treated with first‐line pembrolizumab versus chemotherapy [11, 12]. The National Comprehensive Cancer Network also recommends immunotherapies (e.g., durvalumab) as consolidation therapy following definitive chemoradiotherapy in unresectable stage II and III lung cancer, with significant survival benefits [10]. These advances, along with improvements in access, have resulted in greater numbers of patients receiving immunotherapy treatments [13] and improvements in survival times observed in people with lung cancer [11, 12].

The immunotherapy toxicity profile differs significantly from that of chemotherapies [14]. Chemotherapy is associated with a broad range of cytotoxic side‐effects that impact multiple body systems, including haematological and gastrointestinal. Typical haematological toxicities include anaemia and neutropenia, while gastrointestinal side‐effects frequently include nausea, vomiting and diarrhoea. Neurotoxicity, such as chemotherapy‐induced peripheral neuropathy, is also common. In addition, patients often experience fatigue and pain, whether following surgery or as a result of radiation‐induced oesophagitis [5]. By comparison, the most common side‐effects for patients receiving immunotherapy are immune‐related and include pneumonitis, fatigue, pain, muscle aches and joint pain; with other common symptoms including rash, dry skin, dyspnoea, cough, diarrhoea and constipation. More severe symptoms are generally reported by participants with poorer performance status [15]. However, it can be difficult to predict which patients will experience the greatest toxicities [16].

There is growing interest in the efficacy of rehabilitation interventions [17], in particular exercise, to counter the toxicities experienced with immunotherapy [18, 19]. Exercise may offer a potential mechanism to modulate the tumour microenvironment and enhance the body's response to immunotherapy; mobilising T and natural killer cells, improving cytotoxic immune function, and reducing levels of inflammation [20]. There is currently a paucity of evidence in lung cancer regarding the safety, feasibility or efficacy of rehabilitation during and following immunotherapy. Participants of most lung cancer rehabilitation trials to date have been managed surgically or during and post radiotherapy, chemotherapy or, to a lesser extent, targeted therapies [21].

In the context of this emerging and under‐evidenced area of practice, ensuring that rehabilitation interventions are acceptable and feasible to patients, carers, and healthcare professionals becomes particularly important. It is established that patient experiences are associated with clinical safety and effectiveness [22]. Experience‐based co‐design (EBCD) is a participatory action research approach which has most frequently been used in the redesign of health services. This methodology involves partnering with end‐users to increase their engagement in intervention design [23, 24, 25]. The EBCD process may assist in ensuring the needs of both those participating in and delivering lung cancer rehabilitation programmes are met [26]. EBCD commonly involves patients, carers and healthcare providers or other identified key stakeholders working together to re‐design services, with a focus on storytelling and the user's experience [23, 24, 25]. Examples of the use of EBCD methodology to identify issues (referred to as ‘touchpoints’) that influenced care experiences and to re‐design medical services are reported in head and neck [27] and breast and lung cancer [28] populations and in the emergency department setting [29].

This study aimed to use an EBCD approach to (a) understand patient, carer, and healthcare professional perspectives of the lung cancer immunotherapy treatment journey and (b) identify key design requirements of a rehabilitation programme for people with lung cancer receiving immunotherapy. The co‐designed rehabilitation programme will be piloted in a subsequent study.

## 2 Materials & Methods

This study designed a lung cancer rehabilitation programme using a modified EBCD process involving three distinct stages with patients, carers and exercise healthcare professionals. To facilitate the involvement of people from regional and rural areas, the study was conducted entirely online. Consolidated Criteria for Reporting Qualitative Research guidelines were followed in the conduct and reporting of this study [30]. This study received ethical approval from the University of Melbourne (ID 2023‐26609‐39687‐4).

### 2.1 Participants

Participants were recruited through advertisements to patient/carer and healthcare professional organisations, via email to steering committee contacts and on social media platforms. This method meant the total number of people approached was unknown.

### 2.2 Inclusion and Exclusion Criteria

- Age ≥ 18 years;
- Willing to participate in an online individual interview and/or two online co‐design workshops;
- Sufficient English language skills;
- Able to provide informed consent; AND
- People diagnosed with lung cancer (independent of stage) who were receiving or had previously received treatment, including immunotherapy (e.g., nivolumab, pembrolizumab, durvalumab, atezolizumab) alone or combined with other treatments, or their family carers; OR
- Exercise healthcare professionals (physiotherapists, exercise physiologists or allied health assistants) who currently or had previously provided prehabilitation or rehabilitation for people with lung cancer.

### 2.3 Measures

At the commencement of the project, sociodemographic and clinical characteristics (patients and carers only) and prehabilitation or rehabilitation experiences were collected from all participants via a survey. Particular patient characteristics of interest included disease stage, treatment regimens, gender, education, and area of residence. Healthcare professionals were additionally asked about their qualifications and practice setting. Qualitative data were collected during online individual interviews and workshops. Field notes were taken during the workshops to ensure all planned discussion topics were covered; these notes were not included in data analyses.

### 2.4 Procedures

#### 2.4.1 Consent Process

Written informed consent was obtained from all participants for all uses of data described in this report. Consent was reaffirmed verbally at the start of each recorded individual interview and each workshop.

#### 2.4.2 Details of Data Collection, Processing and Analysis

**Figure 1:**
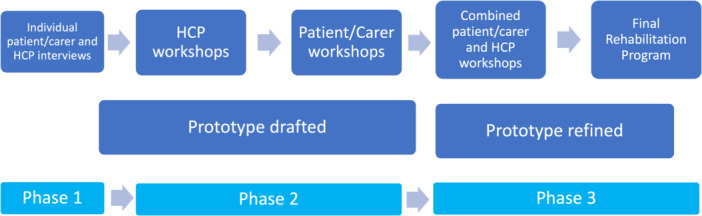
Study stages.

A summary of the key stages of the project is provided in Figure 1 below.

Participants completed the pre‐interview or workshop survey, mentioned previously, electronically via the study's Research Electronic Data Capture (REDCap) database.

**Phase 1.** Individual interviews with patients, carers and exercise healthcare professionals.

**Purpose:** To identify experiences of lung cancer immunotherapy treatment and rehabilitation, alongside barriers and enablers to rehabilitation participation and delivery that would inform co‐design workshop development. Interview schedules were designed by the research team. Questions around rehabilitation barriers and enablers were guided by the Theoretical Domains Framework (TDF) of behaviour change [31]. The TDF is widely used and comprises 14 domains influencing behaviours: knowledge; skills; social and professional roles and identities; beliefs about capabilities; reinforcement; intentions; goals; memory, attention, and decision processes; environmental context and resources; social influences; emotion; and behavioural regulation [31]. The interview schedules were piloted with three patients and one exercise healthcare professional before commencing study recruitment. The interview schedules were revised following feedback (Supporting Information file S1). Data from these pilot interviews were not included in the analysis.

Before each interview, participants received the interview schedule to allow time to reflect on potential answers. All interviews were audio‐recorded, and patient and carer interviews were additionally video‐recorded. Member checking was conducted by sending each participant a written summary of their interview, giving them the opportunity to add or correct information before data analysis. The lead author (LE) edited the patient and carer video‐recordings to produce a 10 min summary of key findings, referred to in ECBD methodology as a ‘trigger film’. Patient and carer participants from Phase 1 were then provided with the trigger film and invited to give feedback, including requests for revisions, before it was used in the Phase 2 co‐design workshops.

**Phase 2.** Separate online 2 h, small‐group workshops involving patient/carers and exercise healthcare professionals were held. It was not a requirement of the participants in this phase to have been involved in the Phase 1 interviews. An overview of the workshop structure is provided in Supporting Information file S2.

The exercise healthcare professionals workshop commenced with participants being provided a summary of the key themes from the exercise healthcare professionals' individual interviews. Participants were able to discuss and revise the Phase 1 themes during the small‐group workshops. Themes were presented in categories according to the Consolidated Framework for Implementation Research (CFIR) [32]. The CFIR is used to evaluate factors which can influence intervention implementation. The five CFIR domains, comprising multiple constructs, include 1. Intervention characteristics, e.g., complexity, adaptability; 2. Outer setting, e.g., policies, external incentives; 3. Inner setting, e.g., structural characteristics, readiness for change; 4. Characteristics of individuals, e.g., knowledge, self‐efficacy, and 5. Implementation processes, e.g., activities used to implement the intervention. Not all themes from the Phase 1 interviews were included. Themes related to the Outer setting and Implementation processes ‐ such as management structure, referral processes, funding, and appointment systems ‐ were excluded. The research team determined that these organisational or external factors were context‐specific, could not be controlled for, and would not be evaluated in the subsequent pilot trial. As such they were considered by the research team outside the scope of this co‐design project.

The patient and carer workshops commenced with participants viewing the revised trigger film and discussing any emerging issues. Emotional mapping was used to explore participants' experiences of rehabilitation during and following immunotherapy by eliciting positive and negative emotions to identify what was working well and what could be improved in the current system.

In each of the exercise healthcare professional and patient/carer workshops, participants discussed priority areas for service change and key design requirements of future rehabilitation services, to enhance enablers and reduce barriers. This information was used by the researchers to develop a draft rehabilitation programme.

**Phase 3.** Both stakeholder groups came together in a combined 2 h online, small‐group workshops. The workshops began with the revised trigger film and presentation of the key themes and draft rehabilitation programme arising from the Phase 2 workshops. The groups worked collaboratively to iteratively refine the final rehabilitation programme.

After each workshop participants were given the opportunity to provide feedback via a REDCap survey about the workshop itself and whether they felt their ideas were listened to, any ideas they did not get a chance to discuss during the workshop and suggestions for subsequent workshops.

### 2.5 Research Team and Reflexivity

All interviews were conducted by the lead author (LE, she/her), a PhD‐qualified physiotherapist, who was unknown to patient and carer participants prior to study commencement. The lead author (LE) had formal training in EBCD methodologies from the Point of Care Foundation. The workshops were facilitated by one co‐author (EP) and the lead author (LE). EP is a PhD‐qualified occupational therapist with experience working with the study population and in conducting group workshops online. They were not known previously to the patient and carer participants. Some healthcare professional workshop participants were known to both researchers who conducted the interviews and workshops through professional networks. Three of the four members of the research team are PhD‐qualified physiotherapists with expertise in lung cancer rehabilitation, which may have introduced pre‐existing assumptions regarding care delivery. All interview and workshop interview guides were developed a priori, and data were coded independently, to reduce the influence of the research team on the co‐design process and rehabilitation programme development.

### 2.6 Data Analysis

Sociodemographic, clinical characteristics and rehabilitation experiences were reported descriptively as mean (range) for continuous variables and counts (percentages) for categorical variables. All interview and workshop data were transcribed verbatim using Zoom and cross‐checked by one researcher (LE) for accuracy. Qualitative interview and workshop transcripts and feedback surveys were analysed independently using content analysis [33] by two members of the research team (LE and EP) to identify key themes of lung cancer immunotherapy and rehabilitation experiences and priority areas for change in the design of the new programme. NVivo 20 (QSR International) software was used for qualitative analyses.

## 3 Results

### 3.1 Participants

**Figure 2:**
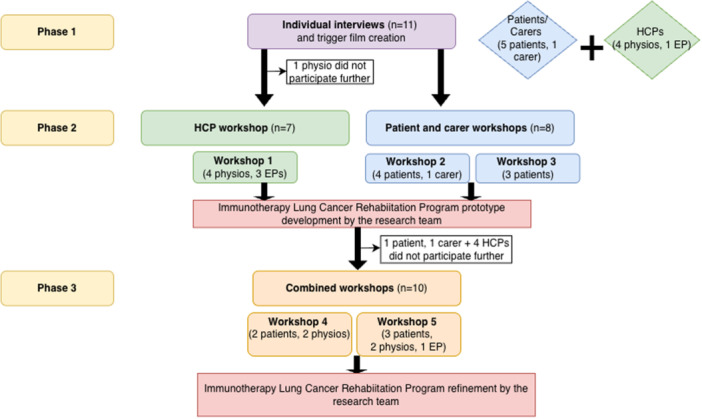
Participant flow. *Note:* Not all participants were involved in Phase 1. One physio participated in Phase 1 and Phase 3. Abbreviations: EP = exercise physiologist, HCP = healthcare professional.

Throughout the study 17 stakeholders were involved: seven patients, one carer and nine exercise healthcare professionals. Data collection occurred between May 2023 to March 2024. Figure 2 illustrates the participant flow through the study.

In Phase 1, 10 individual interviews with 11 participants were conducted, comprising four patients, one patient/carer dyad, and five exercise healthcare professionals. In Phase 2, 15 participants were involved in three separate stakeholder group workshops: Seven exercise healthcare professionals, seven patients and one carer. Phase 3 involved two combined stakeholder workshops with a total of 10 participants: five healthcare professionals and five patients. Seven of the Phase 1 and 2 participants did not complete Phase 3 as they were either unavailable for Phase 3 workshops (*n* = 5), did not respond to invitations (*n* = 1), or were unable to continue due to deteriorating health (*n* = 1).

Most of the patient participants were female (87.5%, *n* = 7/8), with stage IV disease (71.4%, *n* = 5/7) and had commenced immunotherapy treatment more than 2 years earlier (57.1%, *n* = 4/7). Just over half (62.5%, *n* = 5/8) resided in metropolitan areas [34]. Most of the exercise healthcare professionals were also females (88.9%, *n* = 8/9) and physiotherapists (66.7%, *n* = 6/9). Almost half reported more than 5 years of lung cancer rehabilitation experience (44.4%, *n* = 4/9). Over three‐quarters were working in metropolitan areas (77.8%, *n* = 7/9). Table 1 provides further details of demographic, clinical, and professional characteristics by stakeholder group.

**Table 1:** Participant demographic, clinical, and professional characteristics.

Patients and carers (*n* = 8)	*n* (%)
Age, years (average [range])	65 (55–74)
Sex, Female	7 (87.5)
Language (preferred), English	8 (100)
Aboriginal or Torres Strait Islander, Yes	0 (0)
Area of residence^a^	
Metropolitan	5 (62.5)
Regional	0 (0.0)
Rural	3 (37.5)
Education	
Some high school	2 (25.0)
Completed high school	2 (25.0)
Technical and further education	1 (12.5)
Postgraduate university degree	3 (37.5)
Employment status	
Employed full time	1 (12.5)
Employed part‐time	2 (25.0)
Retired	5 (62.5)
Lung cancer histological type (*n* = 7)^b^	
NSCLC	4 (57.1)
Unsure	3 (42.9)
Disease stage (*n* = 7)^b^	
III	1 (14.3)
IV	5 (71.4)
Unsure	1 (14.3)
Time since diagnosis, months (*n* = 7)^b^	
7–12	1 (14.3)
13–24	2 (28.6)
More than 24	4 (57.1)
Lung cancer treatments in addition to immunotherapy (*n* = 7)^b^,^c^	
Chemotherapy	6 (85.7)
Radiotherapy	5 (71.4)
Surgery	1 (14.3)
Rehabilitation participation (*n* = 7)^b^	
Current formal programme	2 (28.6)
Previous formal programme	2 (28.6)
No formal programme	3 (42.8)
Exercise Healthcare Professionals (*n* = 9)	
Age, years (average [range])	39 (29–69)
Sex, female	8 (88.9)
Area of residence^a^	
Metropolitan	7 (77.8)
Regional	2 (22.2)
Rural	0 (0)
Qualification	
Physiotherapist	6 (66.7)
Exercise physiologist	3 (33.3)
Time working in lung cancer rehabilitation	
Less than 1 month	1 (11.1)
13–23 months	1 (11.1)
2–5 years	3 (33.3)
More than 5 years	4 (44.4)
Practice setting	
Public hospital	7 (77.8)
Private hospital	2 (22.2)

*Note:* All values are *n* (%) unless specified. Australian Government, D.o.H., Disability and Ageing. *Modified Monash Model*. 2025 [cited 2025 05.02.25]; Available from: https://www.health.gov.au/topics/rural-health-workforce/classifications/mmm.

Abbreviation: NSCLC, non‐small cell lung cancer.

^a^ Area of residence according to the Modified Monash Model (MMM) geographical classification systems [1].

^b^ The carer participant (*n* = 1) is not included in these variables.

^c^ Participants could select multiple response options.

### 3.2 Phase 1—Individual Interviews

Individual interviews were an average duration of 53 min (range 32–95, total duration 530 min). Themes and participant quotes related to experiences of immunotherapy and rehabilitation, and enablers and barriers to rehabilitation are provided in online Supporting Material 3 and presented in summary in the paragraphs which follow.

Patient and carer participants described significant variations in experiences of receiving immunotherapy treatments.

I really haven't had any bad reactions to the immunotherapy. I've had, you know, small things like a little bit of the tingling in the fingers.

(Patient 1)

And after about 10 min I started going really weird, and I felt really strange and um tearing at my clothes, and I was boiling hot, and I just felt terrible, and they had to stop it…I wasn't breathing very well. I just couldn't get a breath… it was quite hard to, you know, to… live your life when you can't breathe properly.

(Patient 3)

Breathlessness, skin irritations, and fatigue were commonly reported side effects. However, these side effects were generally more tolerable during immunotherapy alone compared to when it was given in combination with chemotherapy. This observation was also reported by healthcare professional participants.

The main thing with immunotherapy, I see is the fatigue and the skin irritation they're the big ones that have. Yeah, that I guess are the most common.

(Healthcare professional 4)

Patient and carer participants felt the purpose of rehabilitation for people with lung cancer receiving immunotherapy included to prevent deconditioning; improve knowledge, fitness, muscle strength, breathing control, lung capacity, energy levels, mood and quality of life; create a sense of purpose and routine; allow engagement in usual household tasks and family roles e.g., playing with grandchildren; increase their ability to tolerate future medical treatment and to increase survival.

I needed instruction. … when breathing became really difficult, they taught me how to bring it under control, and me not expect them to give me a pill.

(Patient 2)

…with each few days I could see that I could do more, or I was getting a little bit quicker getting around the house or walking down to the dam…And I wasn't getting as breathless as I was at the beginning.

(Patient 18)

I feel on such a high every time I go do my exercises and come back. But it really does give me a really good mental, happy mental frame of thinking… or mood…

(Patient 1)

And I think that if my cancer should come back and I have to do treatment again well if I'm fitter then it's not going to be so hard on my body because I was pretty fit when I first got the cancer.

(Patient 18)

Rehabilitation information was lacking, and access to services was mixed. Experiences ranged from participants who had been referred to and participated in formal hospital outpatient programmes, to those who had not been able to access services and had exercised independently and unsupervised at home.

I looked on the internet. I even spoke to the hospital. There was a nutritional fellow that you could go to but other than that. No, I couldn't. I couldn't find anything.

(Patient 18)

Exercise healthcare professionals reported limited training opportunities.

There hasn't been too much from an allied health [perspective],…. training programme in immunotherapy…. So any programme like that I would do, whether it was a webinar or something.

(Healthcare professional 7)

Numerous enablers and barriers were identified by either healthcare professionals, patients/carers or both stakeholder groups. Enablers commonly reported by patient or carer participants included having supervision and support from suitably qualified exercise healthcare professionals, family, friends and peers, and experiencing improvements in mood, energy levels, fitness and quality of life following exercise.

it's always good to have um someone to… fine tune things, perhaps, or you know, to say, ‘well, if you straighten that back leg more’, or you know ‘if you took bigger steps, or smaller steps on the treadmill’. You know that kind of fine‐tuning thing is useful, I think.

(Patient 3)

I certainly feel energised after it. And I was very, when I wasn't doing it, I was very tired.

(Patient 3)

The other people in the programme, listening to them, their experiences, what they've gone through.…. They were so encouraging. We helped one another, just talking and chatting and walking out the door…

(Patient 2)

My 5‐year‐old granddaughter…. I couldn't play with her you know, because it would just take all my breath away, and she would start doing little walks with me and things like that…

(Patient 18)

Healthcare professionals reported enablers including having dedicated funding and appointments allocated to programmes, which allowed flexibility around medical treatment schedules, having the required facilities and equipment and receiving referrals from the multi‐disciplinary team who had received education about programmes they could refer to.

adequate, FTE [full‐time equivalent, staffing]. Often when I talk to people they'll want these things and they'll have a staff member running from somewhere for 2 h. The only way I can go and go ‘Oh, I've got 5 min. I'll just pop out and see [name] on chair 8’,… It's because there's been an appropriate amount of FTE allocated to the service.

(Healthcare professional 7)

We're in a full, fully equipped gym, which is, we're pretty lucky. Lots of space.

(Healthcare professional 4)

And that's that's with any new service. It's gonna take a little bit of marketing, you know, but oncologists they'll see one patient doing really well, and they'll think oh, maybe these other ones will benefit from it.

(Healthcare professional 4)

Barriers to rehabilitation reported by patient and carer participants were: time and travel commitments, costs, a lack of social support, symptoms including breathlessness, fatigue and low mood, deconditioning, fear of exacerbating symptoms, lack of confidence to exercise and poor technology skills.

if you had to go in, go in and do it, but it's a lot of driving in and back to do say half an hour, or an hour of exercise.

(Carer 17)

a lot of the programmes where you need private insurance to access them. Many people can't. A lot of people can't afford private insurance.

(Patient 3)

Because I was scared if I pushed myself too much, I was scared that I wouldn't be able to breathe because I'd get so breathless that I would go too far, and I'd fall over and die, and that's the truth.

(Patient 18)

Barriers raised by healthcare professionals included a lack of time and high workloads, the burden placed on patients caused by additional appointments, a lack of managerial or organisational support, lengthy and non‐automated referral processes and a lack of staff to support telehealth services.

running groups is a bit of a you know. It's a bit of a burden, because you still got to then, you know, let's say, Hey, you get an hour to be able to do a group. You actually don't. You need a bit longer, because you need to be able to do the actual you know the charts and things afterwards.

(Healthcare professional 7)

The patient's buy in, to also then go, ‘I've got lots of appointments. If this is not a standard part of my pathway. I've got too many other things. I've got too many appointments. I've got to go for scans. I've got to go for blood tests. I've got to go for this.

(Healthcare professional 5)

We then also hit the roadblock of admin. So the way patients are referred to us at the moment, it is an electronic referral system. … They get put into the admin system. They then get physically printed out. They get put in a tray which we've got to physically pick up, physically triage, physically put back in a tray, to then be inputted by the admin assistants to then be contacted, and offered an appointment…

(Healthcare professional 5)

Table 2 summarises enablers and barriers reported by participants during individual interviews, using the CFIR domains. Some were concepts identified as being both an enabler and a barrier, e.g., the presence or absence of exercise supervision.

**Table 2:** Enablers and barriers to lung cancer rehabilitation identified during interviews.

CFIR domain	Theme	Enablers	Barriers
Outer setting	Organisational factors	Education of referrers	Lengthy referral processes
		Managerial support	Lack of managerial support
		Student help with workload	Workload
		Dedicated FTE and appointments	
			Organisational structures and priorities
		Doctor encouragement to participate	
			Patient costs ‐ program, transport, parking
			Lack of services
Intervention characteristics	Program structure	Group classes	
		Accessibility	Travel distance
		Supervision	Lack of supervision
		Peer support	
		Flexible class times, delivery modes	
Inner setting	Program content	Outcomes demonstrate program value	
		Facilities and equipment	Lack of equipment
		Individualisation	Lack of individualisation
		Clinician encouragement	
		Enjoyable and varied exercise	
		Feedback on performance	
		Help with technology	
			Irrelevant education
Characteristics of individuals	Individual factors'	Clinician knowledge of immunotherapy	Limited clinician training
		Rewards: seeing patients improve	Lack of time
		Adherent patients, Committing to the program	Patient appointment burden
		Treatment experiences	
			Carer actions
			Stigma
		Improved fitness, independence, mood, quality of life, side‐effects, energy, treatment tolerance	Breathlessness, Fatigue
		Family, friend support	Lack of social support
		Fear of deterioration	Deconditioning
		Financial rewards	
		Financial situation	
		Habit formation	Unfamiliar habits
		Pacing	Fear of symptom exacerbation
		Telehealth access	Poor technology skills
		Weather	Weather
		Planning	
		Prior experience	
		Prioritising exercise	
			Poor mental health
			Reduced self‐efficacy

*Note:* Text colour indicates enablers and barriers identified by healthcare professionals (in blue), patients/carers (in green) or both groups (in orange). Cells in enabler and barrier columns are blank when a matching enabler or barrier was not identified.

Abbreviation: CFIR = Consolidated Framework for Implementation Research.

### 3.3 Phases 2 and 3—Workshops

The study workshops each ran for an average of 1 h and 37 min (range 1 h and 33 min to 2 h, total duration 536 min).

#### 3.3.1 Programme Structure

Hybrid centre‐based and with telehealth options would be the ideal mode of delivery to increase accessibility. Group rather than individual exercise was preferred by most due to the social support provided. However, one male patient participant expressed the desire to exercise independently at home through the performance of daily tasks around the home. Ideal programme durations ranged from 8 to 12 weeks. Session durations of 1–1.5 h would allow time for education and social interaction. Programmes should include tapered ongoing ‘check‐in’ support for the months immediately after completion of supervised sessions to facilitate transition to exercising in the community. Flexibility around programme times was felt to be essential due to treatment and clinic appointments.

#### 3.3.2 Programme Content

In terms of what content should be delivered, individualised and supervised aerobic and strength training exercise was preferred. The importance of including support to change exercise behaviours, including through the use of goal setting, was noted. Several education topics were highlighted as being current areas of unmet need, with people wanting information from trustworthy sources. Topics included fatigue and breathlessness management, the benefits of exercise and how to assess exercise intensity, the common side effects of immunotherapy, eating well and wellbeing.

#### 3.3.3 Programme Facilities and Equipment

A welcoming, suitably sized, quiet and well‐equipped space was deemed essential. Equipment included pulse oximeters, stationary cycle and treadmill, weights machines/free weights/Theraband and ways to monitor adherence and exercise intensity (e.g., use of a traffic‐light system for people to rate how people are feeling on the day and how hard they want to work during the session). Having support to troubleshoot issues with telehealth, especially with larger group sizes, was noted as being essential.

#### 3.3.4 Programme Healthcare Professionals

With respect to who should be involved in the delivery of rehabilitation, most people felt that exercise clinicians, e.g., physiotherapists and exercise physiologists, with oncology‐specific experience were required. There was concern about exercising in a gym‐setting where instructors may or may not know the specifics of a person's current disease or treatment side effects. Screening for additional needs, including nutrition and psychology was desired, although the limitations in accessing some of these services were noted.

### 3.4 Post‐Workshop Surveys

After the Phase 2 separate stakeholder group workshops, two healthcare professionals and two patients provided further feedback via survey completion. This included the addition of education regarding nutritional and psychological supports, longer‐term follow‐up, broadening of education from ‘fatigue’ to ‘symptom management’ and involving additional staff, e.g., nurse or care co‐ordinators. The additional healthcare professional responses indicated they felt there was a consensus reached for most points and good discussion within the group, which was well facilitated. Patients fed back that exercise needs to be tailored to the individual in terms of needs, ability and motivation. Additionally, it was felt that patients should be educated close to the point of diagnosis that exercise would play a big role in their recovery and toleration of treatment, whilst acknowledging the stress of being newly diagnosed and the amount of other information patients are given about their treatment at that time point. One consumer, who had been receiving immunotherapy for several years, indicated they would like exercise reminders to keep moving and encouraging their lungs to be at their best. For this participant, monthly information was preferred as they did not want the rehabilitation programme to take over their lives.

Anything will be fabulous, as up to now nothing has been offered, we had to look/Google and seek help… getting the worst stuff most times, it's unreliable, extreme and most probably not current. Our medical teams don't have the time for all our what seem like silly, inconsequential questions, that mean so much to us.

(Patient 2)

#### 3.4.1 Final Rehabilitation Programme

**Figure 3:**
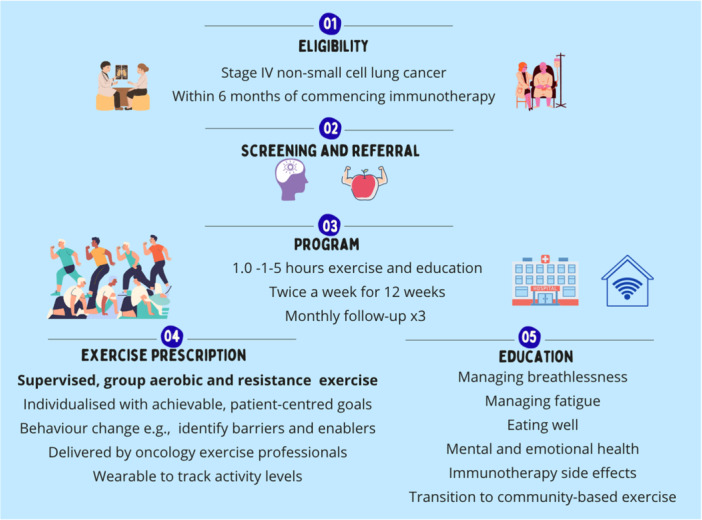
Final co‐designed rehabilitation programme.

Following refinements to the programme during the combined workshops, the rehabilitation programme co‐design was completed. A single survey response was received after the combined workshops, indicating the programme was a very good summary of what had been developed during the previous workshops. Key rehabilitation programme elements presented in Figure 3 were:

- Eligibility which allowed patients to commence within 6 months from starting immunotherapy, once the initial busy/stressful time had settled a little.
- Screening for malnutrition, anxiety and depression, with referrals made based on screening results.
- A 12‐week group exercise programme with flexibility to perform this either in the hospital gym or at home.
- Exercise prescription, which is individualised and progressed or regressed as required; with the use of wearable devices to track physical activity.
- Education incorporating breathlessness and fatigue management; available either online or in hard‐copy depending on patient preference.
- Monthly check‐ins and linkage to community programmes following programme completion.

## 4 Discussion

Through a co‐design process with patients, carers and exercise healthcare professionals, this study has outlined key elements for a rehabilitation programme suitable for patients with lung cancer who are receiving immunotherapy treatments. Individual interview themes included immunotherapy side effects were generally more tolerable than chemotherapy, access to rehabilitation was lacking and limited training regarding immunotherapy is available for exercise healthcare professionals. The key elements of the programme include individualised aerobic and strength exercises, screening for nutrition and psychological support needs and supervision by exercise professionals with oncology‐specific experience. The programme should have flexibility to enable completion in hospital, community or home settings at a range of times to accommodate busy medical review and treatment appointment schedules.

Co‐design methodology has been used to develop programmes in a wide range of populations, but has only recently been applied to lung cancer rehabilitation. An Australian study reported a rehabilitation programme developed for people with operable lung cancer used co‐design, and several themes aligned with those identified in the current study [35]. These included the need for flexible, individualised interventions, multidisciplinary input and unmet educational needs. While the needs of patients with early‐stage disease having surgical resection may differ to those with advanced disease treated with immunotherapy, these concordant themes suggest core rehabilitation programme elements that may be required across a broader range of treatment types. Unlike the previous study, the majority of patients in this study had experiences of receiving chemotherapy and radiotherapy, as well as immunotherapy; with a number having been managed with concurrent chemoimmunotherapy for several initial treatment cycles and then moved to immunotherapy alone as a maintenance therapy. Aligning with previous reports in the medical literature [14], the patients in this study reported significantly higher symptom burden during the combined treatment, with symptoms becoming more manageable following a period of maintenance therapy. These considerations highlight the essential requirement for programme individualisation and careful monitoring with progression and regression of exercise programmes in line with symptom fluctuations. It must also be considered that people with lung cancer and known genetic mutations (e.g., EGFR, ALK, ROS‐1) are commonly managed with targeted therapies which, given their differing mechanism of action, are associated with a different side‐effect profile than the patients involved in this study [36].

Co‐design of health care systems and programmes that better meet the needs of both health care professionals, patients and carers can result in greater applicability, acceptability, uptake and adherence to programmes than those designed by healthcare professionals alone [26, 37]. Despite the rapid increase in the use of co‐design methodologies, particularly in Australia [37], studies which compare health outcomes or cost‐effectiveness of co‐designed studies to outcomes of more traditionally designed studies are lacking [26]. A rapid review of co‐design studies indicates that evaluations frequently include stakeholder perceptions of the process [26]. Patient stakeholders often report positive experiences, such as increased confidence managing their own health conditions and enhanced skills for contributing to research. Researcher stakeholders commonly describe benefits in terms of strengthened collaboration with community groups [38]. However, challenges are also noted, including greater time and costs associated with co‐design processes, tension between stakeholder groups regarding the maintenance of research rigour and negative experiences where involvement has been perceived as tokenistic [38].

Registered randomised controlled trials of exercise interventions for people with metastatic lung cancer receiving immunotherapy that have completed recruitment include Hi‐AIM (NCT04263467) and ERICA (NCT04676009). Both these trials involved centre‐based, supervised high‐ intensity interval training for time periods of 6 and 13 weeks, respectively [39, 40]. However, neither of these interventions was co‐designed. ERICA is investigating feasibility as its primary outcome [39, 40], with Hi‐AIM's primary aim being changes in levels of circulating natural killer cells [40]. Qualitative research findings to date, and findings from this co‐design study, indicate centre‐based exercise training may not be the model of exercise intervention that is most acceptable to many people with lung cancer [41, 42]. The rehabilitation programme co‐designed in this study offers flexible delivery through a hybrid model combining centre‐based and home‐based settings. Both exercise healthcare professionals and most patient and carer participants emphasised the importance of supervision across these settings. This approach helps balance stakeholder preferences for location while preserving key elements of effective exercise prescription—such as supervision [43] and intensity [44] —that are known to enhance health outcomes.

A limitation of this study is the low proportions of males in both patient or carer and exercise healthcare professional groups. This imbalance may affect the generalisability of the programme, particularly given established gender differences in exercise preferences [45] and broader cancer care needs [46]. Evidence showing males tend to prefer independent, outdoor activities, whereas women value supervised exercise, support and the social connection offered by group‐based programmes [45], is consistent with the findings in this study. Likewise, the number of participants living or working outside metropolitan areas was small. This occurred despite online methods for data collection that aimed to reduce barriers to participation for people in regional or rural areas. The healthcare professional group of only exercise professionals could have included additional experts such as lung cancer clinical nurse consultants, dietitians, psychologists or occupational therapists, who may have provided different views. Further, budgetary constraints prevented inclusion of participants who did not speak English, limiting the programme's generalisability to this disadvantaged group. It will be important to understand how appropriate the resultant programme is for males, non‐English speaking people and people living outside major cities. The use of technology in this study may also have created a barrier to participation for some, although support was provided prior to the workshops for participants who expressed a need for assistance to join and participate in the online sessions. Guidelines for the conduct of online co‐design have been published since the completion of this study, which can be used to support future online co‐design studies [47].

## 5 Conclusion

This co‐designed rehabilitation programme for people with lung cancer receiving immunotherapy is currently being pilot tested at a cancer centre in Australia. Importantly, the study has highlighted the impact of different and changing medical treatments on patients' abilities to participate in rehabilitation. The co‐design process is anticipated to enhance the programme's acceptability by aligning its structure and delivery with the needs and preferences of patients, carers and exercise healthcare professionals. Pilot testing will evaluate feasibility, acceptability and safety and may provide evidence to support a larger hybrid trial of effectiveness and implementation.

## Author Contributions

**Lara Edbrooke:** conceptualisation, methodology, formal analysis, data curation, writing – original draft, writing – review and editing, visualisation, project administration, funding acquisition. **Elizabeth Pearson:** methodology, formal analysis, data curation, writing – review and editing. **Catherine L Granger:** conceptualisation, writing – review and editing, funding acquisition. **Linda Denehy:** conceptualisation, writing – review and editing, funding acquisition.

## Ethics Statement

The study was conducted in accordance with the Declaration of Helsinki and approved by the Ethics Committee of the University of Melbourne (ID 2023‐26609‐39687‐4, approved 09/05/2023).

## Consent

Informed consent was obtained from all participants involved in the study.

## Conflicts of Interest

The authors declare no conflicts of interest.

## Supporting information

Supporting file 1_UNITE_Interview_guide.

Supporting file 2_Workshops_outline.

**Table S1:** Immunotherapy experiences – data from individual interviews. **Table S2:** Rehabilitation experiences – data from individual interviews. **Table S3:** Enablers of rehabilitation. **Table S4:** Barriers to rehabilitation.

## Data Availability

The data presented in this study are available upon reasonable request from the corresponding author.
