# Supplementary material for: Co‐Designing Lung Cancer Rehabilitation Services for People Treated With Immunotherapy
Source: Health Expect. 2026 Apr 5;29(2):e70660. doi: 10.1111/hex.70660 (PMC13052158; doi:10.1111/hex.70660)
Supplement: Supplementary file 1 — Supporting file 1_UNITE_Interview_guide. [file HEX-29-e70660-s001.docx]

UNITE study: Individual Semi-Structured Interview Topic Guide

**Topic guide for interviews with consumers –**

1. Can you talk me through your experiences of being on immunotherapy treatment for your lung cancer.

Prompts – Where are you at with your treatment currently?

How have you found the immunotherapy treatment?

*[prompt as to whether they have had any symptoms during or following treatment]*

What do you think is the purpose of pre or rehabilitation for people with lung cancer before, during or after immunotherapy?

1. Can you talk me through your experiences of participating in pre or rehabilitation?

Prompts – When did you commence pre or rehabilitation in relation to your immunotherapy treatment?

What did *[does]* the pre or rehabilitation program involve *(disciplines, duration, delivery mode, exercise components, group versus individual)*?

1. I’m going to ask some questions now about what may have influenced your ability to participate in pre or rehabilitation before, during or after your immunotherapy.

Where there any challenges in participating?

Was there anything that helped you participate?

Prompts:

1. Did you feel you had the skills to be able to perform your pre or rehabilitation program? What supervision or education did you need?
2. How confident were you that you could perform your pre or rehabilitation program?
3. During your regular daily routine did you expect that you would be able to perform your pre or rehabilitation program?
4. How did your pre or rehabilitation program fit with your priorities of what you had to get done every day?
5. Did you ever do anything to make it easier for you to complete your pre or rehabilitation program? What did you do?
6. What do you think would have happened if you did not perform your pre or rehabilitation program?
7. What, if any, rewards did you receive from completing your pre or rehabilitation program?
8. How much effort did you go to, to complete your pre or rehabilitation program?
9. Was it ever difficult to complete your pre or rehabilitation program?
10. Can you think of anything that helped you to complete your pre or rehabilitation program?
11. Did other people influence whether you completed your pre or rehabilitation program?
12. Did your feelings ever influence whether you completed your pre or rehabilitation program?
13. Are there any key elements of the pre or rehabilitation program that you think may help people participate? (e.g., length of the program, times offered, telehealth versus face-to-face, group versus individual)
14. Are there any changes to the program that you could suggest that may help people participate?
15. Is there anything else that we haven’t discussed today that you would like to tell us about?

**Topic guide for interviews with healthcare professionals–**

1. Can you talk me through your experiences of delivering pre or rehabilitation to people with lung cancer during and following immunotherapy?

Prompts – What does your pre or rehabilitation program involve (disciplines, duration, delivery mode, setting, exercise components*, group versus individual*)?

When do your patients generally commence the pre or rehab program in relation to commencing their immunotherapy?

What do you think about pre or rehabilitation for people with lung cancer who are having immunotherapy?

1. I’m going to ask some questions now about what may have influenced your ability to deliver pre or rehabilitation to people with lung cancer during or after immunotherapy.
2. Did you feel you had the skills to be able to deliver the pre or rehabilitation program? What training did you need? What future training would you like?
3. How confident were you that you could deliver the pre or rehabilitation program?
4. During your regular work routine did you expect that you would be able to deliver the pre or rehabilitation program?
5. How did the pre or rehabilitation program fit with your priorities of what you had to get done every day?
6. Did you ever do anything to make it easier for you to deliver the pre or rehabilitation program? What did you do?
7. What do you think would have happened if you did not deliver the pre or rehabilitation program?
8. What, if any, rewards did you receive from delivering the pre or rehabilitation program?
9. How much effort did you go to, to deliver the pre or rehabilitation program?
10. Was it ever difficult to deliver the pre or rehabilitation program?
11. Can you think of anything that helped you to deliver the pre or rehabilitation program?
12. Did other people influence whether you delivered the pre or rehabilitation program?
13. Did your feelings ever influence whether you delivered the pre or rehabilitation program (e.g., stress of workload, frustration with telehealth etc)?
14. Are there any key elements of the pre or rehabilitation program that you think may help clinicians deliver it? (e.g., staffing, equipment, telehealth versus face-to-face, group versus individual)
15. Are there any changes to the program that you could suggest that may help clinicians to deliver it?
16. Is there anything else that we haven’t discussed today that you would like to tell us about?
