# Supplementary material for: Co‐Designing Lung Cancer Rehabilitation Services for People Treated With Immunotherapy
Source: Health Expect. 2026 Apr 5;29(2):e70660. doi: 10.1111/hex.70660 (PMC13052158; doi:10.1111/hex.70660)
Supplement: Supplementary file 2 — Supporting file 2_Workshops_outline. [file HEX-29-e70660-s003.docx]

**Workshop Outlines**

The stakeholder workshops were held virtually. An overview of timetable and schedule is provided below to indicate the general format.

| **Phase 2**  **Participants - Healthcare professionals (physiotherapists, exercise physiologists, allied health assistants)** | |
| --- | --- |
| **Aims:**   - Gain an understanding from participants of their clinical experience of providing lung cancer rehabilitation for people during/following immunotherapy - Identify and prioritise key rehabilitation program design elements/areas for change | |
| **Duration** | **Discussion point** |
| 15 mins | Welcome, consent process, plan for workshop and introductions |
| 15 mins | Project overview, aims and plan for the workshop |
| 45 mins | Project team present key themes from the preceding healthcare professional individual interviews  Whole group discussion and activities to review and revise themes (may include small group discussion/activity) |
| 30 mins | Whole group discussion regarding priority areas for change/key design elements of the future rehabilitation program |
| 15 mins | Thanks, next steps and close workshop |
| **Post-workshop action:** project team to transcribe and analyse workshop data for presentation in workshop 3. All participants to be sent a questionnaire and the opportunity to provide feedback regarding the workshop process and suggestions for the combined HCP and consumer workshops. | |
|  | |
| **Phase 2**  **Participants - Consumers** (patients and caregivers) | |
| Aims:   - Gain an understanding from participants of their lived experiences of participating in lung cancer rehabilitation during/following immunotherapy - Provide feedback on rehabilitation prototype developed following workshop 1 (this will also be available for participants to review prior to the workshop). - Identify and prioritise additional topics, high level re-design and revision of content of, or inclusion in the rehabilitation prototype | |
| **Time (approx.)** | **Discussion point** |
| 15 mins | Welcome, consent process, plan for workshop and introduction |
| 15 mins | Project overview, aims and plan for the workshop |
| 45 mins | Project team to play consumer participant trigger film  Whole group discussion of any emerging issues and ‘emotional mapping’ exercise to reflect on the emotional impact of the trigger film touchpoints |
| 30 mins | Whole group discussion regarding priority areas for change/key design elements of the future rehabilitation program |
| 15 | Thanks, next steps and close workshop |
| **Post-workshop action:** project team to transcribe and analyse workshop data for presentation in workshop 3. All participants to be sent a questionnaire and the opportunity to provide feedback regarding the workshop process and suggestions for the combined HCP and consumer workshops. | |
| **Phase 3**  **Participants - Healthcare professionals and consumers** | |
| Aims:   - Participants will work together to prioritise areas for change/key design elements of the future rehabilitation program which were identified in the phase 2 workshops. | |
| **Time (approx.)** | **Discussion point** |
| 15 mins | Introductions, project update, aims and plan for the workshop |
| 15 mins | Project team to play consumer participant trigger film |
| 60 mins | Whole/small group discussion and activities regarding joint priority elements/changes of a lung cancer rehabilitation program |
| 30 mins | Thanks, next steps and close workshop |
| **Post-workshop action:** All participants to be sent a questionnaire asking for feedback regarding the workshop process. The project team will work to integrate key design features into the future lung cancer rehabilitation program with workshop participants surveyed about this and feedback used to revise the program to be piloted. | |
