# Supplementary material for: Co‐Designing Lung Cancer Rehabilitation Services for People Treated With Immunotherapy
Source: Health Expect. 2026 Apr 5;29(2):e70660. doi: 10.1111/hex.70660 (PMC13052158; doi:10.1111/hex.70660)
Supplement: Supplementary file 3 — Table S1: Immunotherapy experiences – data from individual interviews. Table S2: Rehabilitation experiences – data from individual interviews. Table S3: Enablers of rehabilitation. Table S4: Barriers to rehabilitation. [file HEX-29-e70660-s002.docx]

Table S1. Immunotherapy experiences – data from individual interviews

| **Theme** | **ID** | **Quotes** |
| --- | --- | --- |
| **Patient and carer participants** | | |
| **Immunotherapy experiences** | **3** | my oncologist, just started me on it er after I had radiation I had to wait a little bit. I had radiation to the brain and I had to wait for a little while after that and I had been an inpatient for a bit and I did this as an outpatient. I had to have um a port put into my arm, |
|  |  | I had to do the immunotherapy, and, as I said, it wasn't as scary as some of these other things, but I went, and they I already had the port in my arm, and they plugged me in and I had my daughter with me. It was okay for a minute or 2. And after about 10 min I started going really weird, and I felt really strange and um tearing at my clothes, and I was boiling hot, and I just felt terrible, and they had to stop it. Um this is a very first time I had it and then, they had to give me steroid, and they gave me antihistamine and other things, and then er then we continued it after that. Yeah, you have to understand it’s every 3 weeks. So I I was a little bit more nervous the next time I went after the first reaction. And so they gave me steroid cover. At the beginning of it they they gave me some steroid before I started um and then the next time um I didn't have the steroid cover, and I was okay. And then, after I don’t know, I don't know how long after it was. It might have been with by the time the next one was due. I wasn't breathing very well. I just couldn't get a breath, and my husband's a medico, and he said, ‘Oh, maybe you need to go and see a chest physician. So I did, and he didn't know what it was. He thought maybe I had COPD, or you know I wasn't breathing. And then they did this thing, and they said, ‘Gosh, you lungs have got look like someone with Covid. They've got like this pattern in them’. And and I said ‘it's not pneumonitis ?’ Because I'd read about it on the information sheets from from the Keytruda that it says you can get any itis you can get gastritis, colonitis blah blah blah. And I said ‘it wouldn't be that from the Keytruda?’ And it was. So I basically diagnosed myself, and with this, and I had to then have steroid treatment at home. They were coming and giving me, plugging me in, again to my port at home. Hospital in the home. I don't know how long I had the steroid therapy for, probably 4 or 5 days with an infusion pump. And that was alright. They just left the pump here and came back and re-plugged me in but I think it wasn't very good for my hip all of this, really. |
|  |  | And then I sort of got over that, and I started to be able to breathe a bit better. But they said my lungs were quite scarred, functionally, sort of not as good as they were um yeah. |
|  |  | the actual immunotherapy was kind of minor in the scheme of things and I and I still had to stop it, because I I had like some grade of of pneumonitis that they said they couldn't really continue |
|  |  | it was quite hard to, you know, to to live your life when you can't breathe properly. it's like having bad asthma or something, you know. I couldn't get enough breath, |
|  |  | it's complicated, because then I got the hip replacement. I you know my hip went. I thought I've been hitting a golf ball too hard, and and I, so I got a very funny weird feeling in my hip that was like quite painful when I walked and did things, and I had a scan, and they said, ‘Well, I'm very sorry you've got avascular necrosis, and that's from the steroids that you've been on’, and I went, ‘Oh’, ‘and you have to, and and you have to have a replacement’. So that became that was the object of the rehabilitation. Initially, not the lungs. |
|  |  | I was very tired. I got very tired, and I don't know what it was from. I, I there's nothing wrong with my bloods or anything like that. So I don’t know. |
|  | **16_17** | Well, [participant name] started having chemo and immunotherapy together and initially, he had four big doses of 2 chemotherapy drugs. And the immunotherapy and then it went to every 3 weeks chemo immunotherapy. But then he got a temperature which, as a you know, typical male, refused to go to hospital until I absolutely insisted, because, you know, they they said that was an emergency. |
|  |  | he had a rash all over him. He felt well, but he just just had this temperature going up and down, you know, over a number of days. And so then they determined. Really, the long and short was, we'll have to stop the chemo, but we'll keep going with the immunotherapy. But meanwhile, in the background, before that incident occurred, there was also some concerns, because his liver and kidney results from the blood tests were being impacted by by them as well by the 2 therapies and so you know they were monitoring that and there was a couple of times where we would go in, and then they would say, ‘Look sorry. Your your results aren't good enough for us to proceed. You’ll have to go home’, [laughing] and so we go home and then do a blood test next week. |
|  |  | So since he's being just on immunotherapy, I'm doing all the talking. But he's he's actually, he's actually felt a lot better because he he wasn't really able to get out like we live on an acre of land. So we've got a lot of gardening, and we're trying to subdivide our block. So it was right at a point where we needed to be doing masses of work, and we couldn't do anything, you know. So there was a lot of pressures behind the scene for us as well. So now he gets out, you know. He gets tired, and you don't do as much as you used to, but he's still getting things done |
|  |  | Well, I feel I’m becoming a bit more useful. |
|  |  | but with the immunotherapy that’s had its complications as well and of course we were worried that either. So his kidneys have since he started. All this. His kidneys have progressively had a reduction in the functioning quite a bit. But so they referred us to the nephrologist to to see what, to determine what that might be the cause of. So [participant name] had blood pressure. That's been well managed. But during a lot of this process it wasn't well managed. It didn't seem to matter what happened. He was having quite high blood pressure and they tried different drugs and stuff, and one of those drugs caused his legs to swell up, which is a common side effect, apparently |
|  |  | So the nephrologist has said, she's feeling pretty confident that it's the blood pressure that's causing the kidney issues and the oncologist has said he'll have a pretty low threshold, but he feels that there’s every chance, you know, based on the nephrologist’s assessment that we will continue with the immunotherapy for the next what was due for another year. and for us, which is being great benefit. He's gonna make it every 6 weeks instead of every 3, and double the dose cause, he said. It still still has its efficacy. There's no major side effects compared to you know what you'd experience, anyhow, and that's just gonna make life a lot easier for us as well to only come in every 6 weeks instead of every 3. |
|  |  | UNITE16: plus the immunotherapy like, I can go in by myself, you know, ‘cause it doesn't doesn't bother me. UNITE17: You do get a little bit tired. UNITE16: Yeah, I do get a little bit tired. UNITE17: He gets a little bit tired after 4 or 5 days. But generally, you know, he's not seeing major side effects are you? |
|  |  | people don't realize, I think, is the impact on your whole life. And and it's awful to say that really when someone's got cancer, and they're thinking that, you know, will I survive this. But you know this, this running parallel with that is the financial and other impacts that it has, you know |
|  |  | Interviewer: How long, [participant name], do you think it was after you've sort of stopped with the chemo that you started to feel a bit more useful again, and have less of those side effects from the chemo? UNITE16: 3 or 4 weeks. The first week after chemo, I basically lay on the couch and just watched television. I might have got up and washed a dish, but I didn't do much, didn't do much at all. And I think, too, because I used to work a lot. The the cancer stopped everything. |
|  |  | So I become, and not like I just became like nothing |
|  |  | Because I’m confused about the whole thing. that one retiring and I've got got really sick. I couldn't do things. I felt useless. Well, why should I feel useless? Because I'm sick |
|  |  | That that came with a degree of fear, I'm going to say, because the whole idea of 2 years every 3 week maintenance of 2 drugs. And then suddenly, well, we've got to stop this one. And so it’s like what does that mean, you know? Because that mean you're at a higher risk of the cancer returning because you're doing it to stop microscopic cells landing somewhere and growing? And you know. The oncologist said, look the the first 4 big ones you had probably did the major stuff. and you know so who knows. I don't know. They they do that, I suppose. To be sure, to be sure, maybe. But yeah, anyhow, we just got to take it as it comes no good killing you trying to have chemo |
|  |  | There's there's a significant difference. Yeah. You like, you go out there. You're out for an hour, 2 hours, and he's got to stop and rest for quite a while he'll have. If he sits down he'll fall asleep. you know. Even looking after the grandchildren. |
|  |  | Oh yeah, well when I mow the lawns our nature strip is huge, probably about 50 metres by 100 metres, but it's on a slope so I have to use the push mower. I'll say to [participant name], ‘I nearly get a heart attack every time I do it. I used to do that before I went to work. you know, because it didn't bother me. Now it bothers me. You know we've got what we call the good lawn near our house area. I do with the hand mower and collect the clippings and yeah I'm tired at the end. So there's a lot of you know. I did some concreting and I’m tired. |
|  |  | Oh, well, I guess I guess things like you know, there was times you couldn't drive. It's the fatigue, you know. I couldn't trust [participant name] to drive into the city cause. He couldn't stay awake. But just with the immunotherapy that's being progressively got better like this twice now he's just got in on his own because I couldn't go, for whatever reason |
|  | **18** | Firstly, because I'd only had a couple of weeks break between from my radiation and chemotherapy. The the actual experience was a lot better, but I had no energy. There was. There was just nothing in the tank whatsoever. um to the point of couldn't stand long enough to have a shower. There was just nothing left in the tank |
|  |  | By the afternoon I'm I'm really tired |
|  |  | 100 percent. The side effects on my immunotherapy were nothing compared to chemo or radiation. Because it's so hard because I only had that 2 week break to tell what the symptoms were from whether was whether it was from the immuno or the other. If I had to say just immuno. I only felt funny, and it is not a specific thing that I can say I just didn't feel right, and that would last for about a day. And then I was fine, the actual, I didn't have any reactions to the immunotherapy |
|  |  | I did 6 weeks of chemo and radiation every day for 6 weeks, and then I had a 2-week break. Then I started my immunotherapy for 12 months. |
|  | **1** | Yes, I I think really, it's maybe been a positive. I really haven't had any bad reactions to the immunotherapy. I've had, you know, small things like a little bit of the tingling in the fingers. I certainly haven't had a rash, maybe an occasional red dot here and there. But overall I I haven't had any bad reactions or something, that's yes stopped me from my general activities to life. The only thing they think they have, which is what I'm being treated for at the moment. I'm on steroids because of a cough, and they think that that the cough and congestion I've had is maybe a side effect from the immunotherapy. |
|  |  | I was first diagnosed January ‘22 with the lung cancer. So then we went through the process of obviously the doctors needed to decide how they would treat me and then so we went through chemo and radiation and then, after that period I went on to immunotherapy. So maybe not quite a year, already. |
|  |  | So at the moment it when I was having, is every 3 weeks I would have the treatment |
|  |  | I had an infection in the lungs but it continued on, and that's when they looked at. maybe it was a reaction to the immunotherapy, a side effect. |
|  |  | So I do have a problem with continence, yeah. Anyway. So the cough really did affect that, because if I was drinking water to relieve it. so we had lots of fluid. so yes, it did have an impact |
|  |  | But it was annoying for me, I'm sure, for my yeah, my husband it drove crazy. We're very conscious of covid. So you know your cough, and you're explaining that it's not covid. So there are impacts. you know, just in your day to day living aside from the fact that you're coughing and yeah. |
|  |  | Because one of the things the oncologist said to that because I had radiation, there could be scar tissue in there. So all of that possibly was contributing to the cough as well. |
|  | **2** | it’s been amazing, I’ve had little or no side-effects and the ones I might’ve had I’ve forgotten about. Which has been really good. I may have had a bit of prickly skin in your hotspots like in your armpits and under your breasts or in bed of a night, a bit agitated. But I can't think of anything else that I would classify as um a complaint um I really can’t it’s just been superb for me |
|  |  | last Wednesday. I had treatment Number 110 so, but in the beginning of immunotherapy I was having it once a fortnight, and then, after I think 12 months. They said, well, you've tolerated it so well. It's working so well. you know the cancer is shrinking back, we're gonna drop it to once a month. So I think I’ve been on immunotherapy for 6, 6 years, this June around about this June because I was on chemotherapy to start off with had a round. And was off for 6 months and then it came back. So that's when they started immunotherapy. And it's 7 years since my, since I was diagnosed. So I’m going to say 6 years I’ve been on it. |
| **Healthcare Professional participants** | | |
| **Immunotherapy side effects** | **4** | And I think on the whole patients, in my observations they do tolerate it a lot better, but it maybe throws up a few different side effects that we you don't think of when you think of radiotherapy or chemotherapy. So sort of skin irritation and that sort of the one that springs to mind that I see with most of the patients it's the most common |
|  |  | The main thing with immunotherapy, I see is the fatigue and the skin irritation they're the big ones that have. Yeah, that I guess are the most common. |
|  | **6** | And we can, you know, and then obviously if they are having their immunotherapy, and they are quite fatigued you know, we just pull the program back a little bit, make it a bit more gentle, so that we can manage just monitoring their fatigue as we go, so that they’re coping with treatment, but at the same time encouraging them to continue to come to us to exercise. |
|  |  | very similar program to pulmonary rehab and just again, just watching, I watch. I try and watch the fatigue for everybody, but especially for the ones undergoing the treatments. |
|  | **7** | We don't, we don't see the neuropathies in the immunotherapy that we do in chemotherapy. So often the only limitation to a patient’s walking might be comorbidities like osteoarthritis |
|  |  | And you can be talking about digestive function. You can be observing skin reactions, you can be looking at oedema and some of these things. So when it comes to the early stages of of ipilimumab particularly and nivolumab when it comes to looking at exaggerated immune responses and things like that, if if you built the rapport with the patient, and you spending an hour with them in a supervised exercise setting. You're talking to them about dyspnoea, you're talking to them about all sorts of things. |
|  | **8** | So most of those I guess are those that have side effects from their immunotherapy. And probably the 2 most common would be pneumonitis and colitis reasons for admission in those patient populations. |
|  |  | the pneumonitis ones we're generally tackling it a little bit differently, because the shortness of breath is much more severe. |
|  |  | I think it is quite individualized. I definitely couldn't say that, you know, it's cycle 3 for every patient. I think it is generally later in the cycles, because I think and it'd be great to see what the consumers think. But I think my experience is the lung cancer patients really have a lot of hope for their immunotherapy and then told that it's better tolerated than chemo. So I think a lot of patients will actually hold off coming into hospital. So it tends to be a little bit later. They’ll generally try to wear those side effects generally people don't think like coming into hospital, no matter what their diagnosis is. So yeah, tends to be a little bit later. But I think, individualized. |
|  |  | And it and I think the patients are really receptive to it, because the ones that come in with side-effects they're feeling really awful. You know, they're breathless, or they've got really bad colitis and stomach pain, and that's when we're trying to deliver this education to them about exercise and I just don't think the timing is ideal for them. |
| **Immunotherapy impact on survival** | **7** | nivolumab used early in public health care and there are a few patients to date that are, you know, still alive and and and thriving very well, appreciating the the services that were extented to them at the time of their diagnosis |
|  |  | But yeah, given that, they've been on these treatment pathways for it is, you know, very nice to be involved in that you know, chronic management of their lung cancer. |
|  |  | non-small cell lung cancer patients, we are almost managing them a little bit like a COPD patient now, with good lifestyle and good medical management. You know, they're living with and and beyond their cancer quite a lot. |
|  |  | quite concerning language around an initial diagnosis. But then someone with 46 cycles of nivolumab on their Charm. They might reflect and go, ‘Oh, gosh! I I didn't think I'd get to here’. |
|  |  | some of the the response curves with immunotherapy alone, chemo-immunotherapy, some of those curves that we see in in life expectancy, and treatment outcomes |
|  |  | If someone has COPD, you sort of can discharge them. But it but it's it's the whole complexity of. Okay, I'm just. I've got nivolumab four weekly and I just keep going. And and it's that kind of like you. You just don't like, you know, when when you are working with those non-small cell lung cancer patients that have had that many cycles, they get to a point with their oncologist they’re like, ‘do you want to stop? Do you want to have a break? When do we start up again? What do we do,’ like there's this sort of ambiguity around like ‘And my cured am I treated?’ |

Table S2. Rehabilitation experiences – data from individual interviews

| **Theme** | **ID** | **Quotes** |
| --- | --- | --- |
| **Patient and carer participants** | | |
| **Experiences of Rehabilitation** | **3** | I wasn't offered any because I wasn't having trouble really um I was having other troubles, you know like when you've had brain surgery, it's a little bit difficult. And I had to have home help, and for a little while, because you don't bounce back that fast. So I did have home help, and I had an exercise physiologist coming around at that point to the home. |
|  |  | And I think, my GP suggested I I do that. Now she may have actually recommended this this person who came to my house and they gave me a chronic care management plan. |
|  |  | I had a terrible, terrible limp er and I couldn't walk upstairs, and I was quite uncomfortable. Couldn't play golf. Couldn't do anything um and he suggested Keiser. |
|  |  | Look it, it costs a lot of money, you know, because I'm doing this privately the GP did give me some more sessions, but I think it's you know, it's insignificant, really [laughing]. So there's not much funding for that sort of stuff, but not that I expect it. I don't. You know, I can manage to pay it myself, but it's just a bit of a I'd probably do more if it wasn't so expensive. |
|  |  | a lot of the programs where you need private insurance to access them. Many people can't. A lot of people can't afford private insurance. |
|  |  | I don't think that GPs really, know what to do. They, I mean, I had to ask to, to get referred to someone, I had to ask. I said to my GP, ‘well, look apparently I can get advanced care or not what, not to advance care what's it called chronic” |
|  |  | And I can get some physio or some exercise physiology as a part of that’. She said. ‘Yes’. And she didn't recommend anyone, she said, ‘Well, do you want? Where do you want to go?’ And I said, ‘Well, I'm already going’. And she just wrote it to them. But she wasn't, she didn't give me anything in the community or suggest anything. |
|  |  | Well I got recommended by an orthopedic surgeon because I couldn't weightbear, and he said, ‘Well, you need to do non-weightbearing exercise, you know, like swimming or something. All right, I didn't really feel it was winter again, and I didn't really feel like getting my gear off and doing water aerobics, hydrotherapy or anything. So he said, ‘go try Keiser’. |
|  |  | you need to remember to make a longer consultation if you going to do all these sorts of things. |
|  | **18** | I can remember, remember thinking. Why isn't there a program for people that are going through this that know what to do? |
|  |  | But there was nothing out there, you know, for an exercise program for anyone that had been through lung cancer that I could find |
|  |  | I looked on the internet. I even spoke to the hospital. There was a nutritional fellow that you could go to but other than that. No, I couldn't. I couldn't find anything even to this day. |
|  |  | But if there's some, the first point is being able to access something. There was just nothing that I could find. |
|  | **1** | But it's interesting how I got to be a part of part of that program because of the coughing through my GP. The nurse, we found a physiotherapist who deals with damaged lungs just to see if that would help. So I went to her and she actually also works at the [organisation name] and she referred me to to the Physio part to help me to get started with the exercise |
|  | **2** | I never had rehab. I never had any form of therapy. I had no counselling. I wasn’t put in. I never spoke to a lung cancer nurse. I’ve never um. There’s just been nothing. It's just been go and see your oncologist every month, or for every appointment. And talk to him, and he looks at your blood tests. Go over the road and have your treatment, have a talk to the girls over there. They were all the information I had do it from home. So I’ve had nothing. if I had known what was available, what would have benefited me, until I got sick last year um was when they thought rehab would benefit me |
|  |  | they’re telling me the rehab that I had then and when as an outpatient at [organisation name] for 14 visits had nothing to do with my lung cancer. Nothing whatsoever. (inaudible) to do with the pneumonia and the blood clots in my lungs, nothing to do with my lung cancer at all. |
|  |  | I came home, and I was still unwell, and I knew. And they said, you've just got to get over this. but I wasn’t taught or told how to get over it. It was just go home |
|  |  | Maybe they thought (inaudible) maybe they thought well we don’t have to talk to her you know she's doing it all, anyway. (inaudible) the cancer didn’t knock me around too much. I did as much as I could. Maybe it’s just my fault for not asking. |
|  |  | I knew I was getting sicker and sicker and I couldn’t get anyone to listen to me. They were going ‘oh you’ve had pneumonia, that’s why you feel so crook’. |
|  |  | gave me a list of all like these people I could talk to, I rang them all up to find out how much they would cost me even with the Medicare, healthcare plan and I said ‘you’ve got to be kidding I can’t afford that’. |

Table S3. Enablers of rehabilitation

| **Subtheme** | **ID** | **Quotes** |
| --- | --- | --- |
| **Patient and carer participants** | | |
| **Supervision** | **3** | it's always good to have um someone to, to fine tune things, perhaps, or you know, to say, ‘well, if you straighten that back leg more’, or you know ‘if you took bigger steps, or smaller steps on the treadmill’. You know that kind of fine tuning thing is useful, I think |
|  |  | there were some things, some machines, that I didn't know that I needed help to show to show me what to do on them. You know some arm things, and ditto with with Keiser. I I needed help to to be shown what to do. But once I knew what to do. It's fine. |
|  | **18** | if there was something in place where you were guided through it, or there were, or there was someone there. It would make life a lot easier for people going through it and having to build their lungs back up if you can call it that or their energy levels all that sort of thing. |
|  |  | And like I said, it's it's taken me near on 3 years to get where I am that yeah, it's just determination. But I think the big thing is like having that support person. Yeah, that is, that is there that, you know, if it all you collapse or whatever happens |
|  |  | I probably would have gone there for a few weeks to a month, until I found confident that I could do it at home and then then I wouldn't, wouldn't go again |
|  |  | you're so scared of that. You'll do the wrong wrong thing, or you'll make things worse if there's somebody there or program there to say, well, you know, if you start this off gently, or and and do this and do this. That's a start, and then once people can do that with confidence then I think the majority of the time that they're happy to sort of keep on going forward. Because the the reward for me, the the reward was just amazing. That I got at the end of it all. |
|  | **1** | So yesterday I had aside from [name of HCP] my my supervisor. I had a young lady there, and she stayed with me the whole time. She helped me do the counting, watched what I was doing reminded me. you know this, this exercise you need to put your head down, or whatever. So they, even though we're in a group, they actually do watch what you doing to help you to make sure you do the exercises correctly to achieve the goals. |
| **Prioritising exercise** | **3** | I do prioritize the exercise physiology, because I think it's important. And I just, I just make appointments to fit round |
|  |  | I made it like an appointment I'd go to in the morning, and because I needed to get fitter, and needed to get stronger and you know |
|  | **18** | If I had treatment I would, either because I've always gotten up early, do it in the morning, or if I had an early morning appointment, I made sure I did it of an evening when it was cooler. yeah, and it's not having to leave the house. So it was easy |
|  |  | the determination. Not yet. Yeah, this cancer just wasn't gonna beat me |
|  | **2** | it was really only about 45 minutes a day and I if I couldn’t fit in 45 minutes a day there is something wrong. Um my partner was also stopping in and doing stuff. So, with me. He was doing his own bit so that made it a lot easier for me as well. And I. And every day I think oh maybe today I'll be able to make the bed. and I got to the stage where I’d say ‘Oh [partner name] don’t help me.’ and every day just got better. And my doctor said to me, write a gratitude diary of what you could do today that you couldn't do last week. I didn’t write the diary, I’d forget. But I thought to myself oh gosh you know I managed better than yesterday, without sitting down every half a minute. Um, I could manage to gauge how much better I was getting on a daily basis that way. |
| **Financial situation and rewards** | **3** | probably what's made it easier is that I'm not, you know, I'm not broke. I mean, if I was, if I was a broke pensioner, as many people are, there's no way you could afford this. |
|  | **16_17** | AIA are having sort of rewards if you get certain number of steps a day and stuff like that connected with their app. and I know some friends of ours, that's encouraged them to walk a bit more. So, I thought, oh. I want to change, anyhow, for other reasons, you know, without boring you with those. But I thought that that might motivate us, because there's certain financial rewards in regards to the level of your excess and stuff like that. So I thought, well, maybe that'll encourage us to try and reach the number of steps you know. We need per day to to do that. Yeah, get certain points and stuff |
| **Enjoyable exercise** | **3** | No, it's quite enjoyable. I find |
|  | **16_17** | I love blockey things. I know you know, and doing stupid stuff like that, you know, like I'm not interested in, you know doing hand exercise, you know, and exercises, or anything like that. I'm just not, it just doesn't. I just sort of sit there and go, why? you know Interviewer: so am I right in thinking you you'd kind of prefer to do things that are on your own? rather than like UNITE16: I’d prefer to do things with [participant name] |
|  |  | To be perfectly honest if if I'm if I'm going to do exercise, I prefer to grab the dog and put a leash on him and go for a walk. |
|  | **18** | I got on a push bike and that's, they're an electric bike, but you still have to pedal. and I must say that that is the only exercise that I have done where I felt I had that endorphin thing kick in and feeling really good, that all these people talk about. |
|  | **1** | I really enjoy it. I haven't found it difficult at all. |
| **Improves mood** | **3** | I know it's gonna do me good. |
|  |  | You know I was feeling awful for a while and thinking about funeral music, and you know and I'm not now. So it's obviously made my mood better. And, and I guess if you if you get that, you got to keep it on going. It's a bit self-reinforcing. |
|  | **2** | I felt so much better after I do those exercises, now whether that’s because I actually breath better, or whether it's just the mental tick that I’ve done it and that’s what’s making me feel better I don’t know, um sometimes [inaudible] no it's not hard. We’re retired, we’re retired, what else have we got to do except look after ourselves? |
|  | **18** | Just my mental state. I think. There's things in life I still want to do, travel and things like that. So I knew in myself that if I wanted to be able to do those things I have to get my fitness level up, I have to be able to you know, walk and and and deal with things. You know, it's mentally as well, I think as physical |
| **Family/friend support** | **3** | My husband's quite encouraging and, and my family, you know. I mean, I just say, ‘I’m go to gym, you know, rehab gym but they are pleased that I've got a lot better. So I guess I guess it's a bit of validation from, from my family as well. but also, I mean, it's basically so I’m fairly self-motivated |
|  | **16_17** | But if [participant name] would walk I'd walk, and I,I know that saying that I'm putting it on, [participant name]. |
|  |  | I see my kids once, twice a week every week. I see my grandchildren once, twice a week, every week. They all know me. They all run up to me. They're all happy to see me. And I think for me I'm taking more out of that. Than. like cause, I think one of the oncologists said at one stage, you're saying he had a lot of colds, I said, ‘well, grandchildren’. They said ‘don’t see them as often’. I said, ‘well, I'm not going to do that’. |
|  |  | so my only stimulation now is my grandchildren. |
|  |  | But if if there was something where you could just do things on your own in your own time. That might be more, what would work definitely from what I'm hearing for you, [participant name], is that, have I kind of go that right? UNITE17: yeah but to be honest I think that would require me saying, ‘Come on, [participant name], you've got to do this. Get it done. Come on you know I'll come with you, and you know let's go out and do’. It would require me to have that to give him that push. would you agree? UNITE16: most of the time. UNITE17: Yes, most of the time don't think he'd be self-motivated to be honest, and that's an individual thing |
|  |  | UNITE17: but if if the hospital said you need to do a program, you would. Probably if it was close to home, you'd probably do it, wouldn’t you? UNITE16: Probably. Yeah, I wouldn't. I'd do it grudgingly, but I'd do it mainly because someone would make sure I did it. [laughing] |
|  | **18** | Made sure my husband, was here. and I said, this is what I'm going to do. Can you just watch out. ‘Oh, I I'll walk with you’. And I said,’ No, I need to do it myself. But just be aware this is what I'm doing’. and that's how I got the confidence to build up, because I had. It was like I had a back up. He was there that if something happened and I just slowly built up. |
|  |  | My 5 year old granddaughter...The fact that I've got her and I want to live to see her get older but in the beginning. I couldn't play with her you know, because it would just take all my breath away, and she would start doing little walks with me and things things like that. Yeah, she gave me the motivation, I suppose in in some ways. |
|  | **1** | You know I feel very fortunate that I seem to have a body that responds to treatment and and so um and I think I have great support to help me to get to this point just within the family and my friends. So it it helps you to be confident about you know, taking the next steps and helping yourself. |
|  |  | Yes, when, once I'm at home, I'm gonna be encouraging, dragging, I'm not sure what word I should use, my husband to come with me because, you know he works from home, and you know he needs to get out, too. So it'll be something nice. And we live in an area. We have nice parks and along the [name of creek] we can walk or bike ride. So that's the next goal is that we can do it together to encourage each other I guess |
|  |  | when you're sort of committed, or you know it's the weather’s a bit yuck you can kind of encourage each other once the habits there. And you know, once you really start to really enjoy it and feel the benefits that helps you keep going |
| **Exercise HCP encouragement** | **3** | having very positive, um positive role models, and in that the, the exercise, the EPs that I've been working with are all incredibly um positive and encouraging, non-critical. And they've got endless patience |
|  |  | They’re really. They're good and encouraging, and, and they know you quite well. You know that you get a personal relationship with them. And you know, see you next week, or see, you know, I I think having that relationship is, is good and having, I think they've got to be the right sort of person, because the people at say [name of hospital] I don't think they even knew who I was and they didn't know what I could do, what I couldn't do. So they need to actually have an understanding of what your capabilities are, and where your weaknesses are, and what you need to really work on. |
|  | **2** | support, Whether it be to help you with doing your exercises, talking to you. Today wasn't a good day but next, tomorrow might be. Don't expect you’re going to get better really quickly, you know it may. It's it's working at it. But the support probably more, that you’re not alone. You’re not on your own and there’s I mean everyone says, ‘There’s someone worse off.’ And that’s true um but to look around and take in more than your own feelings, and how you feel, and what you’ve been through that other um, scenario of what other people are doing helped me a lot. |
|  |  | They seemed to understand. I don’t know. Just take your time. You can’t do it now. Don’t say you can’t do it, which is what I was saying, ‘I can’t do that’. You can’t do it yet, just put that word on the end and you will get there. And that gave me, er, I knew I was going to get there because they were going to make sure of it. |
|  |  | When I went to [name of organisation] and [name of organisation] I thought I can’t do this. I just can’t. But I had to because they were putting in the effort and I had to at least try. They were coming and getting me every day and making me go down to the gym. |
|  | **2** | I don’t know about confident but determined. Yes, I did feel confident, but I was determined that I was going to get better, and I was going to use everything I could, anything I could to get back to what I was before or as close as I could to before. And she said I could. She said ‘I’ll do that for you’. So I put all my faith in her. |
| **Exercise variety** | **3** | It's good to have a range of, of things you can do. So it doesn't get boring. It needs to be that that's another thing that needs to sort of engage you, whether it be physically or mentally, because some of the stuff they do is quite it requires balance for coordination as well as strength or cardiovascular. You know, it's it's sort of multi-factorial. It shouldn't, it shouldn't just be boring. I mean, we shouldn't just be repetitive. Because that would stop me going, I think and if I didn't progress as well, so you need to see a bit of progression. |
| **Feedback on performance** | **3** | I watch the exercise physiologist and they’ve got a sheet for every, you know, for every person, and they've write the date and they but you can see the progression, she said to me this morning, ‘Oh, your blood pressure is up a bit today’, and she can look down the list and see what my blood pressure has been, what my maximum heart rate is. And you know all that over time. |
|  |  | getting that feedback about how things have changed as a result of you exercising, kind of rein... UNITE O3: it's motivating a, and it's reinforces the, the value of the, of the whole thing really |
| **Individualisation** | **3** | I can do as much or as little as I like, really. Look if people aren't feeling well. they can sit down, have a rest, or they get a drink of water for you. You know, it's, it's, it's fairly self-paced and you can really go hard if you want to. I think that's important, too. If it's in a group you don't feel you don't feel pressured because it's not, everyone's doing different things. Everyone’s working on different things. So I if that's important, too. I think you're not being forced to keep up with lots of other people |
|  | **16_17** | In some ways I think it depends on the person |
|  | **18** | I think it's which way they would want to by themselves, whether it be individual or in a group. But I think both would work yeah |
|  |  | if hospitals could have a gym or something where there's support people there with the program, or easy access to go somewhere you know. But yeah, having having that um program designed for different stages of where people are. Because if they're like me in the beginning, you you know, you can't do anything until you start building up. but I think to also have support. Have a support, some sort of support there as well, because I think the psychological part plays a big bid in the physical part of doing the exercise and getting better again. |
|  | **2** | We all had slightly different things that we would do but we’d just go off and do our own things which was good. |
| **Doctor encouragement** | **16_17** | UNITE16: if someone's you know, the hospital said. You know it's important, you get more exercise, I'd I'd do it. |
|  |  | UNITE17: I that's what I was trying to get at... you know, instead of it being so much voluntary. If I was saying, ‘this is an important part of your program. We want you to engage in this’. you know. I think they'd be I'm not saying you probably do it all the time, but there'd be more likely you do if you know. |
|  | **1** | my GP, doctors, oncologist was happy, were really happy I was doing this. Like everybody was really positive around me. So I think that really helped, you know. Oh, this is good, you know. yeah. And I think my oncologist is really pleased that, like that I'm a not proactive. I don't know if I'm proactive, but that I'm willing to do these things to help myself. I think that makes him happy. Because he can maybe see that the the time and effort he puts into the thinking about the treatments and what's going on. And it's a lot of hard work for them. And I really appreciate that they care, cares for me, but others as well. So when I see that that's make me happy too. |
| **Pacing** | **18** | I've always been a pretty determined sort of a person. And I listen to my body. Basically, when I felt that I'd had enough, I would stop and I just made sure I didn't push myself too much to to to go over the edge for me |
| **Improved condition** | **18** | But in the beginning I thought I wouldn't be a fifth of what I used to be. so I was pretty pleased with myself, I must say, |
| **Improved quality of life** | **18** | It's more self-satisfaction. I can do a lot more than what I put in the beginning, as in just you general daily tasks of running a household and all that sort of stuff like I said. I don't think I'll ever be back to what I was with the damage that's been done I'm quite happy to say I've got a pretty good quality of life I'm happy with where, where I am And that's due to being mobile and exercising. |
| **Improved fitness** | **18** | with each few days I could see that I could do more, or I was getting a little bit quicker getting around the house or walking down to the dam. It wasn't taking me as long my my speed increased, I suppose. Yeah. yeah. I mean, I can't run or anything like that. But yeah, I did. I did notice that like, I said, my speed was increasing. And I wasn't getting as breathless as, I was at the beginning |
|  |  | every little bit extra that I could do. And then when I got to the point where I could do that with no real effort was a little milestone. Oh, go me, you know. |
|  |  | Each time you do them you'll get, it will get easier, and you'll be able to do it all in one go eventually. |
| **Weather** | **18** | I had to pick the time of the day. Like we've got 36 degrees here today. If I went outside and and tried to walk down to my dam and back I'd be huffing and puffing, whereas in the cool I could walk down there and come back and be fine. |
| **Fear of deterioration** | **18** | I need to do it because I've I've I think subconsciously, I think now, if I don't I'll just go backwards. |
| **Independence** | **18** | for me pride, I guess. Yeah pride. |
|  |  | Yeah, I'm just just being able to do things for myself. |
|  |  | But the pride that I can actually for instance our house is totally tiled so I couldn't vacuum the house in one go. I'd have to do it in stages and then the mopping. Today I can go through and vacuum my house have a break, and then I can go through and mop my house, whereas before it was you know, it would take like tag teaming just to vacuum up my floor. That's where the pride comes in. I, you know I'm back where I can do it myself. Nobody likes to do housework, but I think when you have something, and it's being taken away from where you physically can't do things. Those little things, they matter. |
|  | **2** | The benefits have just been overwhelming. |
|  |  | They said I was going to be in 10 to 14 days, and that was, but then when there was hope to come home. You know, c’mon you're doing all right, you know you're expecting too much of yourself, ’cause I had the psychologist helping me saying well you can’t do this yet and that’s when I thought no if I work to their level they’ll let me go back home. So once I worked to that level and I knew I could come home, well, you know if you can get better little bit. You can get better, better every day if you put the effort into it and I mentally started to feel a little bit better. Not much but I still was angry about the whole thing. |
| **Accessibility** | **18** | once the program is developed, to have easy access for the patient, to be able to go and do that with the support there there has to be the support there. |
|  |  | Or even if the program was made that you could access the program. But it's done in your own home, for instance, depending on what sort of program it is, yeah, I think you need some sort of some sort of support. |
|  | **1** | we don't live far away. So it's not a big inconvenience for me. |
| **Peer support** | **18** | Everything seems to be going online these days. I think that would be a really good idea. and then it's up to the individual if they feel that they need somebody with them while they're doing it. Now, whether it be a family member or or whatever. |
|  |  | Support from people that are going through a similar thing, I believe, would be helpful, because they know that person's basically gone through it. I think that would be huge help. But also support wherever your doing that program. Like, I said, if it was if the hops just say that the hospitals had a a gym with a program that you could easily access that there was support people there, that you. you knew that if something have they trained if something happened, you're okay, because sometimes it's when it's a close member of the family that doesn't always work. |
|  | **2** | lots of really good friends and I had them, them as well and every time I would go and get back they’d say ‘oh gosh you look so much better today’, or ‘we can see a huge improvement’. So I was probably doing it for them as well I didn’t want them saying ‘oh gosh poor [participant name]’ um. So that sort of support |
|  | **2** | The other people in the program, listening to them, their experiences, what they've gone through. How much better some of the were. Some of them had had heaps worse than me, they had like half a lung removed and stuff like that, um broken ribs and that. They were so encouraging. We helped one another, just talking and chatting and walking out the door, and I don't know the whole conversation um was really good. That the whole, the group was good. It was sad to leave. |
| **Session timing** | **18** | I'd have to say that it was during towards the end of my chemo radiation or just after. Yeah, ‘cause for me, the immunotherapy. I didn't have really had that much side effects from the immunotherapy. But it was during the immunotherapy, that I was able to start the process of exercising exercising |
|  |  | My thoughts are that it would be morning. Because even now for me, when we get start getting late on in the day. I'm starting to feel tired. I wanna have a sleep and all that sort of thing. So I, I would suggest morning. |
| **Habit formation** | **1** | They taught me how to work the bike, you know, press the buttons and the timer, so they've taught me how to do that. So I can work on that independently |
| **Help with technology** | **1** | But what I liked is that he, [name of exercise professional], watched what I was doing to make sure I was doing them properly, and gave me that instruction. Because then, when you go away and you're trying to continue to do it. It's obviously better for me if I know how to do it correctly |
| **Planning** | **1** | I think we all need a little bit of education on on health and I like the education about why I'm doing these exercises what they're achieving. Because I can't say the routine of exercise is something I find easy. but I know it's essential. |
|  |  | I feel really well. I just know my body needs help. so yes. So those little things. Because, you know, I'm not good with technology. So those little things actually mean a lot to me in, and then being able to manage what I need to be able to go in there and get on the bike and go. Oh, I can press the button so I can. You know I can help myself. which I think will help me to achieve the goal of keeping going if that makes sense |
|  | **2** | I would just prep myself and I would take a couple of Panadol and take my Ventolin. Make sure I was in a calm state not racing around like an idiot or not 10 minutes before you were due to go up to a doctor’s appointment or anywhere else. Just made sure I got it done early. |
|  | **2** | going to it and being physically involved in that um getting up, getting dressed, putting on a bit of make up, you know, and and going and looking a bit nicer that what you do when you traipse around. You know, no it was much easier going in |
| **Commitment** | **1** | Just just to be organized, like to, I I think making the desire to help myself. Being organized like I have a bag with all the things I need to take my water bottle done. I can just pick it up. |
|  |  | I’m a routine person and I just like to be organized and make it easier. So I guess that's what I've done. |
|  |  | I guess you need to make the effort to commit and to go to listen to what they're telling you. I didn't find it a big effort. But you do have to decide but you want to commit |
|  |  | I knew I needed to do it. So the commitment was. I saw the benefit I guess so it wasn't a hard commitment |
|  | **1** | I could see the potential benefit. And I knew I needed to do it. And and when it when it changes from you need to do, you have to do to this is the benefit, and and you want to do it. I think that that makes it easier when you can change from you have to do it like, it's like a schoolmaster sort of thing, to I I want to do this because it's good for me sort of thing. Let me change this. Yeah, for me I think it’s important to change how I look at why we're doing it. |
| **Flexible delivery modes and times** | **1** | So they gave me a couple of choices of time. To choose to help me to fit in with my schedule. which is really great. And so I looked at my schedule about what I thought I could do to continue because it was making the habit was was the big, big goal for me. So I had to look for a time that I could. When we actually stop doing the program that that time slot, I can continue to use and so I found that really helpful. |
|  | **1** | So obviously there are alternatives and for some people that might be good you know, even if it was on zoom you just commit. It's the commitment. Maybe it's not the location, if if you you know, can do it on Zoom, or you know, do it in a somewhere else I don’t know. I think that's that that flexibility is good. |
| **Prior experiences** | **1** | this is my second cancer, so and like the the other cancer was coming up December, 11 years ago. They're not related. It's just um and it's probably you know from that. I know how important exercise is from, you know. So from past experience. And and I'm sure that's a motivator to to to take out of this this extra curriculum from the treatment that I know is essential and in and I know it is beneficial for me. so that maybe has an influence on why I was keen to to do it when it was offered, and really want to participate and get the most out of it, because it really, you know, exercise helped last time. |
| **Healthcare Professional participants** | | |
| **Students help with workload** | **6** | I usually have, I always have students. So, which is always good. And if they are assessing a new patient, then obviously I'm right, I'm very close by to give them extra support. |
|  | **8** | We do utilize students. And we have students. Sometimes. They're able to see those patients if we're not. But we don't have students throughout the year. |
| **Timing on the treatment continuum** | **4** | I'm gonna say, about at least 10 years specializing in oncology kind of dribs and drabs, and mainly I've been sort of working as an inpatient therapist on acute oncology awards and sort of palliative care. But this year, beginning of this year, we've actually started an outpatient rehab um program for all oncology patients. Um so it is still quite a new service and we are building that up, but have seen lots of lung cancer patients there. |
|  |  | Um so sort of obviously with them being at home, we're able to do a lot more rehab. But obviously, you know, there is still the the, the rehab that goes on when they are an inpatient, if they have to be admitted |
|  |  | about 6 months of outpatient rehab, but years of seeing these patients when they come into hospital. |
|  |  | I've got one gentleman on my list who's kind of fresh to mind. So he was, he had his lobectomy in March, and he was referred then by the the physio that saw him post-operatively on the ward, but it was kind of a case of he's having his lobectomy then he's gonna have his radio therapy. So we're gonna start after his radio therapy. So I sort of met him a couple of weeks ago. So June so that that's probably call it 3 months postop. And that was his choice that he wanted to get through his radiotherapy and our admin team just kind of checks in with the patients, for when they're ready to, to the start. So he'd already, he'd started on his immunotherapy then, but it was more a timing and a convenience thing for him. |
|  | **5** | in the inpatient rehab there. So I, although it was mainly ortho, I would also get some of those patients who had either had sort of brain surgery, or sometimes some lung surgery and things sort of like that. So I would end up sort of seeing sort of some of of those sort of patients and again, at that stage, because of where they were, they weren’t specifically on immunotherapy. |
|  |  | So I think for me personally, my my experiences as a physio is being sort of quite broadly across a lot of different rehabs, and I've also worked in a couple of different rehab units, so seen a variety of sort of presentations, but not not so many that was sort of specifically within that immunotherapy and sort of pre rehab. Which I think probably speaks a lot about the availability of those services. You know as opposed to sort of specific experiences as a clinician. I think it's more that they're just not happening. |
|  |  | We can see them any time that they are on active treatment and when it comes to radiation it's for a period of up to 6 months following that. After that 6 months they then get referred into the most appropriate sort of other outpatient clinic. |
|  |  | He does run a couple of classes tends to be a little bit more for those patients who are so during treatment. |
|  | **6** | as part of our pulmonary rehab program I've always been very keen to include people, most of the people we include with lung cancer are post-thoracotomy people, um post-pneumonectomy, pleuro-pneumonectomies so mostly post op. Although saying that quite a few of our COPD patients also have lung cancer, and are in the throws of having that investigated and and perhaps treated. As far as the immunotherapy goes quite a number of the patients are recovering from surgery and are also having treatment with immunotherapy. |
|  |  | So are you, are you sometimes seeing people prior to starting immunotherapy? UNITE06: Yes Interviewer: or during? Or when would people… UNITE06: definitely, um definitely before, it’s sometimes before, it's sometimes during. |
|  | **7** | In the early years, so through 2013 to 2017 that was largely in rehabilitation. So working with patients towards the end of treatment and on completion of treatment. That intersected with some formal programs with the lung foundation and also working within the health service in their pulmonary rehabilitation. So that's some of my foundations in particularly pulmonary rehab have originated from, from the [location name] here the health service runner program called what it historically was called ‘Lung max’, and it transferred over to more broad pulmonary rehab titling. So I still engage in that program. |
|  |  | My current role from 2017 was moving into the acute and sub acute oncology space here within the [location name] Health Service. I'll I'll reference the [organisation name] but that is just the active cancer center, outpatient chemotherapy and radiation therapy which would then include our targeted therapies, our immunooncology agents, etc. I’ve been working here since 2017, and and my role was employed to to start to capture a lot more prehabilitation. |
|  |  | So I'll see a patient quite early on, you know, as per some of the modelling in the Macmillan publications and those that exist around prehabilitation. And I'll see them sometimes pre cycle one. |
|  |  | So it's it's nice to see people sometimes cycle two where we can reflect and then plan. |
|  |  | has access to my services in in the gym prior to their infusion. |
|  |  | As a as a student I worked in in Queensland Health within their pulmonary rehab then obviously working in in the rehab, like I mentioned earlier, feel quite confident. |
|  | **8** | So I think my perspective is a little bit different in that the bulk of the lung cancer patients I see on immunotherapy are those that require hospital admission. Because it's an inpatient case load. |
|  |  | The other group that I would see and provide rehabilitation to would be those, maybe that are like stage 3 non-small cell lung cancer that are admitted with side effects of their chemotherapy. Often it's neuropathies from the platinum based chemo. And so then, during their admission they are then started on immunotherapy as a second line therapy and probably the third patient, cohort, that I would provide rehabilitation to are the metastatic non-small cell lung cancer patients. And they're normally the patients that are admitted for you know, complications of their metastatic disease. So most commonly bony mets and fractures or brain mets, and, you know, changes in speech, cognition, mobility, falls, those sorts of things. |
|  |  | But I think in an, in an ideal setting, we would be wanting to see patients when they start well actually at diagnosis, regardless of whether it's surgical, chemo or immunotherapy. And I think the way our FTE is set up in our center probably doesn't allow that model to work. We're very reactive in that we only see people when they’re admitted mostly. |
|  |  | And it's quite nice when we do, we get the ones that start immunotherapy on the ward because we're actually able to do that. And it and I think the patients are really receptive to it, because the ones that come in with side-effects they're feeling really awful. You know, they're breathless, or they've got really bad colitis and stomach pain, and that's when we're trying to deliver this education to them about exercise and I just don't think the timing is ideal for them. But it's sort of the only time we have. |
|  |  | Yeah, yeah, I think that's a nice point, because they're getting lots of new information from everything like they're getting education from the nurses around, the pharmacists, the doctors and us as well. So yeah, I find that they're definitely more receptive at that point. |
|  |  | Because then we could potentially deliver that exercise education in a better setting. Not an acute hospital bed and also earlier. And then when the patient comes to the ward they've already had that exercise given. |
|  |  | And particularly if you got more referrals in that early stage. |
| **Clinician knowledge acquisition** | **4** | I know it's been around now for a while [laughing], but it is sort of the newer treatment. I'm still trying to learn about it, and the different side effects that come comes with it. |
|  |  | So sort of skin irritation and that sort of the one that springs to mind that I see with most of the patients it's the most common, and that's when you're exercising, or rehab’ing a patient. That’s that something that you've got to kind of consider and something that sort of, you know it's not a not that you have as much experience with, compared to fatigue or sort of muscle weakness. It doesn't come as natural for physios. |
|  |  | Yeah, yeah, absolutely. I thought I had the skills. I knew I was going to be safe and didn't have any sort of issues. It was more just, I guess my own curiosity. You know I'd like to understand it a bit better. |
|  |  | Yeah, pretty confident. I think that just comes with years, the years of experience. And I think you know, when you've worked with people that are so unwell in an inpatient ward, it's quite nice and reassuring when you get the slightly healthier ones that are at home. And you know, returning to, to daily life. |
|  |  | Because, you know, they do have complex needs, and sometimes things will crop up that haven't happened the week before. so I think to see not necessarily specific training. I mean, that's wonderful. But more just having that oncology experience. |
|  | **5** | where a lot of my experience had been previously was probably also within that acute stage, or that acute post surgical sort of word away from the actual specific cancer care role it had been from that acute sort of side. |
|  |  | I think sort of a I said as a sort of a general role I feel I personally probably sort of need to upskill a little bit more in terms of just understanding a little bit more in terms of the specific effects of, because we know medicine is changing all the time, and newer treatments. So actually understanding the treatments, the effects, and the potential for the side effects from some of them. Just because, again, we know that medicine is advanced quickly in certain fields, so understanding in particular some of these newer trials and things that are sort of happening, I feel I'm quite confident in my own general sort of ability from that. But again, you know, I'm still learning in terms of I think you've always got something to learn. I'm you know, and because I I think that is one of the challenges, me personally, because I work across 3 different clinics. I do have to have knowledge across. And I do feel that it's only when you start to really work and nut down in one particular area that you get really, really good at at going sort of back over that. |
|  |  | And one of the things that you mentioned obviously, was the inspiratory muscle training. And I sort of went. Yes, actually, I need to look into that a little bit more as to who it's appropriate for who it's not appropriate for, so I think I will probably feel pretty confident. But I wouldn't say I was super confident, in terms of some of the additional sort of things that maybe we could add value with, and knowing beyond a lot of the what I would consider sort of regulars to rehab. What are some of those specific techniques that are related to this population that could benefit. So, so that is probably one of those, one of those specifics. So I think understanding the newer treatments. And on this and kind of having a really good understanding of some of those specific interventions. |
|  | **6** | I suppose I've always been interested in it because I did work in the acute cardiothoracic for ages. And so then I became quite interested in these patients |
|  |  | But I think this this is great, that there's immunotherapy, because it gives people the opportunity to have more treatment and a better prognosis. So yes, definitely learning more about it. I'm very lucky, being in a big teaching hospital because there are always ongoing lectures through our respiratory team. So you know it, it is, the knowledge is easily available. |
|  |  | I've been to a lot of European Respiratory conferences, and I always go to stuff on surgery. So I have had, you know, tried to keep up to date by going to conferences, I'd say that's the main thing as well as just local, you know. Local stuff. I've always been quite closely associated with some of the, the nurses, the cardiothoracic nurses, and and they often ask me to speak to the pleuro-pneumonectomy patients and you know, just outside in their support group. So, and, and I always sit in on their lectures as well. So yeah, so it's just ongoing information. I think you absorb information through osmosis don’t you. |
|  |  | And we have a ground round every Friday. Which is just open to the hospital. And that's always the specialist speaking about their particular areas of interest. And often we get the cardiothoracic surgeons and oncologist speaking, so that’s really interesting. |
|  |  | I think it's that experience in, in the, the acute wards in the cardiothoracic surgery. I think that was probably, you know it gave me the interest, but also made me confident, because I knew exactly what they'd been through. I knew how many chest tubes they had in, and I knew what it looked like when the chest tubes were pulled out, what it felt like for the patient, so I think that was really, really helpful. I think it would be very hard to do it if you just came in from this side. And you've never seen a patient in the acute ward |
|  |  | I think probably having that acute experience made it not as hard. So you know, when you see those whopping big scars. I've seen it before, you know. You know you don't go. Whoa, that's a big scar, terrible [laughing]. So I suppose, just experience, you know, with people and trying to help them. |
|  |  | I think it just experience, helps you cope with the different sorts of patients, |
|  | **7** | So I have done, like I mentioned before, some oh, quite a lot of CPD around pulmonary rehab and some of the courses I did, obviously with reputable providers like the Australian Lung Foundation. and some of that was due to implementing phase 3 programs in the community. Some of that was particularly as I mentioned before, and that Lungs in Action program that exists. Obviously early on too there were some attractive courses with large CPD points associated with them |
|  |  | One of one of my colleagues on the on the [location name], [colleague name]. He does a a bit of work in inspiratory muscle training. As time has gone on, I've I've often, you know, picked up the necessary training around pulmonary rehabilitation. |
|  |  | more and more work in acute oncology and so, as tou were looking at some of the information and publications around prehabilitation which initially centered a little bit around cpex testing and and that sort of cardiopulmonary exercise testing and cardiology |
|  |  | some of my mentors which are outside of Allied health. I have done quite a lot of training in you know, immuno-oncology |
|  |  | as a a member of COSA and and going to a lot of national oncology conferences accessed most of my immune-oncology language and understanding through medical and nursing pathways. But I would say, COSA particularly is very inclusive of Allied health, and that's often the most welcoming and and comfortable pathway to to the, the more detailed language around the, the medical and nursing management of patients on these treatments. |
|  |  | we we often know the fundamentals. And we will follow certain protocols |
|  |  | But for someone that may not have the the confidence, or the, or all the nursing supports around them, something like this that can give them some guidelines, which is certainly what people are always seeking. |
|  |  | Yeah, I I I would have to say, you know, support for for growth and when I when I first started here at the [centre name]. They employed to the role but they hadn't thought too much about what the service was gonna look like. So I had some medical oncologists and and directors of the department. Really, you know, putting into the the project their own energies and enthusiasms and motivations, and that really helped get you know, trials and pilots and just experiences under way. |
|  | **8** | I've been working in my area for 10 years now. So I felt confident, delivering the rehab program in terms of having an understanding about lung cancer and traditional treatment on chemotherapy and also drawing from what we know about exercising in other cancer groups like breast and colorectal but definitely immunotherapy, as you would know is quite new and so specific evidence in terms of mode of exercise and and what we're prescribing, there's not a great body of evidence to draw that from so I do feel a little bit more, I guess don't have the guidance in that from the literature as as much as you know, those lung cancer patients that are pre-operative or on chemo. I do work with a really great team, and we've got a really good pharmacist and a really good, 2 really great medical oncologists. So they've been really helpful at sort of providing a little bit of, you know, problem solving within the 3 of us about what we should be doing. But I think it would be great to say more evidence around, you know, exercise specific to lung cancer patients on immunotherapy just to sort of guide a little bit more what I'm doing. But I'm definitely drawing more from, you know other areas. |
|  |  | So I do feel confident in that I’II check the literature, you know, fairly regularly. Even though there's not much there. I feel like I'm trying to stay on top of things as they come out. I feel confident, having worked amongst lots of other tumor streams. But yeah, specifically an immunotherapy less confident I would say, just because of the paucity of evidence. |
|  |  | education is probably the most important and we definitely hear that from our new grads in the hospital that you know, getting education specifically around lung cancer. Specifically around, you know the treatment modalities and how that might change over different stages of lung cancer. I think you'd also have to include exercise with bony mets in that as well, because that's definitely a big fear of our new graduates, understandably, when they have a patient. How do they rehabilitate this person who's got bone mets. |
| **Clinician rewards** | **4** | Oh, just personally, knowing that I've made a difference to patients, and you know they, they get so much out of it, and seeing the progress that they're making and even if they do you know, hopefully not but if they do, how kind of have to come in for something else, you know, they have some reaction to something, they're straight back into it as soon as they can. |
|  | **5** | I think as physios we always do. But I I I think I've come to realize over the years that as physios we're quite needy people we like to. We need to see our patients improve it. you know. |
|  |  | so I think as clinicians in that I'm I think the thing I always sort of say to patients is, we're cheerleaders, you know. We don't actually do the hard work. The patients do the hard work. We just try and put them on the right pathway. So I think anytime, you see, patients, that self-efficacy in terms of them either maintaining that strength or slowing the deterioration. Or, you know, best case scenarios are improving. I think it's always a great feeling, because it's not necessarily that we've done that. But we've empowered the patient to do that. And I think that's where the difference comes in. I think it is. It's seeing those individual changes. |
|  | **6** | Well, I think I I certainly would miss it because I I love these patients. |
|  |  | seeing them improve all their outcomes in a very similar way to other respiratory patients. But I think just seeing their confidence grow and seeing them in many cases return, for some of them return to normal, for others to be able to cope with their ongoing treatment with more confidence. |
|  | **7** | I do know that experienced or seasoned allied health workers are always aware of their key performance indicators and and and a a robust rehab or prehab have program can maintain someone's employment and and ensure that they're potentially working, not only as a therapist, but as a therapist in a patient cohort that they're personally interested in. And that they gain, you know a sense of accomplishment and and job satisfaction from. |
|  |  | I've been passionate about oncology and and pulmonary rehab and and pulmonary, and some of those chronic diseases for the, for the complexities of these patients and and the complexities of pain the complexities of you know, physiology. The the complexities of of the psychology, make it quite rewarding. |
|  |  | Occasionally at a conference I'll get dinner bought for me |
|  |  | I think in some of these situations you can, you can have quite a lot of influence on quality of life and and comfort. And you know whether it be physical or or psychological pain that someone's in. So there's a lot of intrinsic rewards, engaging in in these experiences that people have or or journeys, |
|  |  | So to me it's it's about living in a, in a, in a workplace, and a space that that is full of purpose and and rewarding other than economic reasons. |
|  |  | with oncology rehab and prehab growing, and with the aging population, and and with people living longer on these sorts of things, it does probably present professional pathways and and work opportunities for people that have that have been in it for long enough that they can sort of talk directly to it. |
|  | **8** | our rehab in the home physios are really great at sending us and community physios as well are really great at sending us sort of people's admission and discharge 6 min walk tests and outcome measures. So it is always really nice when you've, you know, referred someone they've been really receptive to the rehabilitation that you've provided, and then you can see, you know, an improvement in their six-minute walk test and or occasionally we, generally tends to be the patients that have gone through pulmonary rehabilitation, and then they'll send a compliment letter. Saying, you know that they loved the program and normally, it's along the lines of you know, I was feeling really frail. I didn't have any confidence, and now I'm confident. And I can, you know, access the community. So yeah, they're really nice to receive. |
|  |  | Yeah, well, it's tricky, because we often don't see them back, which is nice, but they don't come back to tell you that they've done well. Sometimes it's when I'm seeing someone else in the cancer center. I'll bump into them in the clinic and it's really nice to say them looking really well. And when they tell, still tell you that they're doing their exercise program, and they're feeling stronger. Or when the partner or carer you see down there, and they’re really thankful and say, you know, ‘they do the exercises every day, and they're getting better at them’. |
| **Dedicated FTE and appointments** | **4** | Yeah, no, that's absolutely fine. You know, if I'm in outpatient, I've got the full day in out patients. So yeah, it's, it's all, that's all fine. |
|  |  | No, I think, yeah, that's absolutely fine. I think my way, as I said, when we're in the outpatient clinic, that's it. That's all we're doing. We're not having to take phone calls, or we don't have a pager attached to us. So we’re we’re there to deliver therapy and yeah. |
|  | **5** | I think that some of the simple things are is for my senior management, saying. ‘Yes, go for it. We support that you take you know an hour, 2 hours out of. We're gonna give you some protected time, which is to run a rehab pre hab program’. |
|  |  | I know if I said to [clinician name] we're going to do this program, he would speak to those patients and say you're doing this. But we need to have that available to offer to go. |
|  |  | It would then also sort of be so that the care nurse coordinators, and also then, admin team sort of get the referral through and we go, book into group, or book into pre-group screening. You know, it's a half an hour appointment with either Physio or EP. Put it in with whichever one is next available. They'll do a pre screening so that we can do some baseline testing. Then they go into this group, this group or this group. So yeah, that's where I see it could and should work. But yeah, it's kind of having that sort of ability sort of to go ‘No, we are ring fencing this little bit of time for that. |
|  | **6** | So that's my main job. Pulmonary and cardiac rehab. And the chest physio outpatients. So yeah, so that's definitely a priority. |
|  |  | es, my main job that’s right. Unlike, you know, a lot of other hospitals. I don't have to run to ICU and then run back to rehab. I just I'm dedicated to rehab. |
|  | **7** | I like to sometimes refine or or restrict opportunities for patients to sometimes engage in rehab. It puts a bit of value on it as well. I know I'll I'll reference the pulmonary rehab that I work in on Thursdays. And and that's 8 30 in the morning is when the first group participates and they’re often your high performing participants and this is a telehealth based program. Then there's the education component from 9 to 9.30, and then your patients with the reduced performance or age, or whatever, psychology, they'll they'll be in the second group. |
|  |  | So confining, this is the time that we do it. This is the intention and and putting some value on the the the staff that are working in there as well, and the value of this, of this opportunity. |
|  |  | So sometimes with me, saying to patients, ‘You book’, makes them value the appointment and make the appointment. They’ll go, ‘like I, I tried to call, and someone else had booked’, and that makes them see that they're not the only non-small cell or small cell lung cancer patient at the. So then the next week they book and it's the same with my program |
|  |  | So yeah, I've managed the workload with a, sorry [interviewer name], with a with a a structured day that gives me parameters as a therapist as well. This is when I do this, and that's when I tell people that's when I do this. This is when my door is open, and this is where my door is closed. |
|  |  | The final thing, I'll probably say is, you know, adequate, FTE. Often when I talk to people they’ll want these things and they'll have a staff member running from somewhere for 2 hours. The only way I can go and go ‘Oh, I've got 5 mins. I'll just pop out and see [interviewer name] on chair 8’, or you know, it, it's because there's been an appropriate amount of FTE allocated to the service, and I I say that with respect, but certainly sometimes service areas will want a program but they'll never associate enough hours to that. And and colleagues of mine you know proof in that that they'll be here and they'll be there and the, the pulmonary rehab the same [clinician name], who's the HP 5 physio there, 2 days of her week are pulmonary rehab Tuesdays and Thursdays all day, and and that works really nicely. And and people know that. Oh, I called on a Monday, and I got one of the nurses, but they said, [clinician name] will call me back tomorrow or Thursday, and it's that kind of consistency and and appropriate allocation that leads to success. Because I get a lot of people that call me and say, ‘Oh, yeah, we're working here. And we, we're thinking about doing oncology rehab and in those situations that's when the programs have limitations then naturally. |
|  |  | But for example, with the the group that starts at 1030. I've got people showing up at, you know, quarter to 10 but I'm still here, and they might see me leave my clinic room with a patient, and I might say at 1130, ‘Look I I can't hang around. I've got a new patient at 1130’, but you know it, it it it does allow for a little bit of flexibility, because I'm here, or you know I do have that time, so that that would be one thing that I've been blessed with, and and that's why the program is probably as flexible and intimate as it is, because I’m 5 days a week all day in this role. |
|  | **8** | And you've got me during winter. So it's probably I might be a little bit, yes, less optimistic than if it was summertime [laughing]. But I think our FTE is quite limited. So we've got one physio for 31 beds of a mixed haem and onc caseload. So I feel confident if there's a patient who is one of those metastatic non-small cell patients with brain or bone involvement, that I would be able to see them, definitely. But for the patients that are, you know, independent. Don't have any new symptoms of dyspnea or reduced exercise tolerance. And you know, are fairly good from physical functioning, they're a low ECOG score patient. I am not overly confident that I would get to them on a daily basis, just because they're the patients that tend to get deprioritised. |
| **Education of referrers** | **4** | Patient choice. I guess it's a bit probably luck of the draw who they see on the ward I think you know, if patients, patients might you know, this chap also lost a significant amount of weight. So he asked about dietetics input. So we actually got two referrals. So you'd have the pre-existing referral from the physio. That was kind of waiting, you know, in holding. But then, also the nurse manager from his, the outpatient icon clinic. She sent in another referral for him to see the dietitian so that kind of, we had both of them sitting there. So yeah, which is good that you know, 2 people are referring him instead of none. |
|  |  | Yeah, but I feel unfortunately, probably just at our hospital, the pulmonary rehab you kind of think, okay your COPD patients or asthma that, it would only be if you had a very experienced physio who would recognize that the lung cancer patients would still be appropriate. So I think it just depends on who's working on that ward or who sees those patients. And that's something we do, you know, inservice training you know, we recently did an inservice to the whole physio department trying to say, you know, these oncology outpatient referrals don't just have to come from the inpatient oncology wards. You know they can come from the gastric surgery wards, or you know the patient that's had a VATS or a lung biopsy, so you know, things like that. So just trying to, trying to market it as well. |
|  |  | I guess sometimes their surgery might seem a bit more minimally invasive than say a bowel resection, or you know it's not, but it's you know they might, it's not as visible. They might not be left as with as many visible deficits. But I think you know breathing is a pretty important part of life [laughing]. So I think if you not breathing up to your potential, then it can have a huge impact. |
|  |  | It was just more getting the patients in, and I think I had one patient in January, and then, you know, now it's up to kind of you know, fully books 3 days,3 weeks in advance. |
|  |  | And that's that's with any new service. It's gonna take a little bit of marketing, you know, but oncologists they'll see one patient doing really well, and they’ll think oh, maybe these other ones will benefit from it. |
|  | **5** | So we've got a little bit of work sort of to do in in that space in terms of educating what we can offer. That is maybe different to EP Or how we work closely with them. |
|  | **6** | I suppose if people aren’t, if they're not referred. Obviously, I'm not on the ward. So I'm relying on people referring those patients, and I think sometimes it may be a delay in that. So it's in this thing, so that patients not doing well with the patients discharged home, and then the doctor doesn't see them for about 6 to 8 weeks, or whatever it is then, and then they think, ‘Oh, okay, maybe you could have done some rehab, but you didn't get it’, so it might be delaying referral. |
|  | **7** | So clear roles and you know, some, you know ongoing education will always capture new staff coming on board so that people could go, I feel comfortable talking about what exercise, physiology, or physiotherapy or speech pathology is. I feel very confident, referring patients to that. I'm aware of the referral pathways. And I, I feel, based on my time with this person, that this is a a referral that's necessary. |
| **Feasible outcomes demonstrating value** | **4** | But then, now that it is up and running, I think they can see that it's worth it. And we're going to be expanding. So I think the results speak for itself, and the patients are talking to their oncologists and saying how much better they feel. So I think that's, you know, marketing that you can't pay for. So once that you get that snowball effect that's going to be wonderful. |
|  |  | it's considered as a center of excellence for oncology but we don't have a designated rehab program, and there's so much evidence out there now for exercise in all cancers. |
|  | **5** | if we can show, or if we can start doing some of these, and we can see that evidence that actually, patients are sort of doing well, and we're getting those better outcomes. It puts a case forward for us to be able to go, ‘Look these are the outcomes if we put in here now, this is why we need either some FTE to be able to do this or the. You know, or we need to sort of be following along these pathways |
|  |  | around August last year [name of clinician] came up to us and sort of said there’s the lung conference coming up later in the year. I'd love to be able to do an update on the utilization survey, that was sort of done about 5 years ago, and they were looking at what was the uptake of EP for people with lung cancer. And he said ‘I'd love to do an update of that and incorporate physio into it’. |
|  |  | I feel that if we can sort of get evidence of these sorts of things and that it works or we can go hey, look, we wanna be part of these sorts of projects. And hey, is there a chance? We can get some tiny bits of FTE to be able to work on some of these things |
|  | **6** | so we would automatically take them early on, you know when other pulmonary rehab programs would only take COPD, we were already taking the lung cancer patients and because they respond so well to treatment as well |
|  | **7** | And what we've done in the last few months is, look for data that is going to really bolster what we're doing. |
|  |  | The the metabolic demands of of a of a cancer rehab is rehab, and it it can be seen as sometimes being linear. And you can, you can be doing everything to anyone else it would show great results. And then you come up against like this person their their 6 min walk test got worse. And you're like, ‘oh, it didn't get worse, we would expect it to get even more worse than that’. So it's like you, you're talking about negatives all the time. So in the last few months we've actually employed some body composition assessments with SOZO which has historically been used in lymphedema. And I'm I'm really hoping that in you know, the next 6 months or so we're going to start getting some really good data and saying that again you could, you know, that person may have lost weight, but they actually maintained their muscle mass. |
|  |  | that bioelectrical impedance that's quick, and it's easy, and it's modern and patients are really keen. Like some sometimes you do assessments and you see, like, ‘Oh, I don't, I don't really want to do a timed up and go, or I don't really want to do a sit to stand, or I don't really want to do a 6 minute walk test’. But you go like, ‘we've got this new machine. And it's it's going to tell you how much muscle you've got, and we're able to work out what you're basal metabolic rate is so we know how much you need to eat every day, like it's an ipad, and it's quick’. And so I'm really excited to see what that will do. And I I like the fact that we can sometimes talk to that, and we can say, ‘This person came in, and their hydration status wasn't good, their strength wasn't good. They went through four cycles of ipilimumab. They went through 8 cycles of nivolumab and they put on muscle.’ You know. So we're able to talk a bit more to saying, this is why we do strength training. |
| **Flexibility** | **4** | Currently not much, but that will come with the expanding the service. So they’ve got a little bit of flexibility, and we haven't had any problems yet. but it would be great to kind of be able to offer them more. |
|  | **5** | for some people, particularly what we're looking at rehab, it's like they don't want to be back in hospital, you know, once they sort of finish their treatment. They're done. They want to get back on with life. and even delivering telehealth. I think for some people, particularly if they're trying to get back into work. I think that can be a challenge, because they've had huge chunks of time off work. They just want to get back into it |
|  |  | So you know, I think it is keeping that in mind that you know as patients, they are also sort of people who happen to have had cancer. And they are trying to get on with their life outside of it. |
|  |  | And then also the, the individual having the time to, to do, to participate |
|  | **6** | You know, we have certain times during the week, and they can come at the time that suits the best. And so there may be some other patients with COPD, there may be some other cancer patients. |
|  |  | And we try and fit in. Obviously, I try and fit in around their treatment times and so that they just not too fatigued. And we can, you know, and then obviously if they are having their immunotherapy, and they are quite fatigued you know, we just pull the program back a little bit, make it a bit more gentle, so that we can manage just monitoring their fatigue as we go, so that they’re coping with treatment, but at the same time encouraging them to continue to come to us to exercise. That, you know, often improves the uptake of treatment. So yes. So again, it's very. I'm very flexible to fit in with the patient |
|  |  | I think the flexibility to be able to fit in around the treatment times, you know around that. I mean, we're very lucky, and probably all the big hospitals are the same. You know, [organisation name] is literally 2 blocks away. But sometimes we have to put the program on hold just for a couple of weeks, if they can't, if they're not coping with everything. |
|  |  | We try and be flexible and you know, that makes it easier for the patient, I think. |
|  |  | So obviously a rolling program, being flexible. |
|  | **7** | So that allows us to capture patients given the scheduling of their treatments and outpatient appointments. |
|  |  | So they get the choice. And they as as a consumer. And you know, under the appointment burden. I think that is experienced by a lot of oncology patients early on in their experience. We will remove some rigidity, as there is already so many compulsory medical oncology pre-cycle reviews, pathology. |
|  |  | That new patients are being diagnosed and referred in every day and that's why I like a a rolling program for oncology as opposed to a a rigid block program |
|  |  | the oncology is different to the the pulmonary rehab that happens here on the [location name]. That's a that's a block program with the the rigid sort of models of education and and and group exercise of of same prescription |
|  |  | You know, patients and their families are willing to engage in telehealth if travel and distance and appointment burden have compounded a little bit. So I'll extend my services to to phone and and telehealth services as well, and that that is all about building, you know, relationships with the patients that when they are in here. So you know some patients that live a bit further away. I'll I'll engage with them through those forms. But then, when they're having a a cycle of treatment, I'll I'll actually come and just catch up with them physically in the flesh. |
|  |  | So that that again is, you know, I think, why, historically, I've said if this person comes twice a week for 8 weeks they do 16 sessions. You're entitled to 16 sessions in the rolling program as well. And I'm just respecting that, you know, 2 weeks in you got presented to emergency. You were in hospital for a week and a day. I want you to feel like you've missed out, and and that comes back to some statements I make around equity of access. |
| **Managerial support** | **4** | So to get it up and running it did take a little bit of time. We've been pushing for it for years, and I think it's just you've got to get the right people listening. |
|  |  | Just, I think, set up like a bit of a business plan and just show that you know our hospital it's, it's considered as a center of excellence for oncology but we don't have a designated rehab program |
|  | **5** | I think that some of the simple things are is for my senior management, saying. ‘Yes, go for it. We support that you take you know an hour, 2 hours out of. We're gonna give you some protected time, which is to run a rehab pre hab program’. |
|  |  | I think it's kind of having that push from the health service. To say that this is a, this needs to be a standard part of care. |
| **Referrals from the MDT** | **4** | But then, also the nurse manager from his, the outpatient icon clinic. She sent in another referral for him to see the dietitian so that kind of, we had both of them sitting there. |
|  |  | I think just increasing awareness with the oncologist that they initiate the program, um the referral and getting a bit of buy in from them. Because we've had some patients that have been referred, and they come for one session, and they're like, ‘Oh, I don't need this.’ And they, they might not feel they need it, but they could definitely benefit from it. So I think if the referrals, are maybe driven more from the oncologists. Then patients like that, and more likely to, to get involved. |
|  | **5** | So just in general across sort of cancer care services they could. Essentially anybody can refer in. Often it's the, it's the CNC, so the care nurse coordinators who will sort of do that because they have that role where they're having that little bit of actual time to talk with the patient and talk through some of those issues. We do get some sort of through from some of the oncology, oncologists or radiation oncologists. as well depending on the patient as such. |
|  |  | But yeah, essentially, anybody can refer. The general majority is is through the care nurse coordinators, and the oncologists who are particularly interested. |
|  |  | We had to look at who had referred in rather than the tumour stream. But what we found was, is that very few patients had been referred in to Physio from the lung tumour stream |
|  |  | We're gonna give you some protected time, which is to run a rehab pre hab program’. I think it's then also sort of having that I think this one, I think once we have got that, we can then turn to the oncologist and say. ‘I've got this time. Send me a patients, and what I want you to be doing is is saying to those patients is as part of your treatment protocol we refer you through for prehabilitation’. |
|  |  | I know if I said to [clinician name] we're going to do this program, he would speak to those patients and say you're doing this. |
|  | **6** | So they’re referred to us, usually referred to us, usually I would say, by the the nurses who are looking after them. You know the case managing type nurses, either from [organisation name] or directly from the wards |
|  |  | I’d love to see more prehab and I think, I think that the trouble with that is often the surgeon’s see the patient and say, ‘we'll operate tomorrow’, so there's no time for prehab, or they, the patients go back to whoop, whoop. And then they're not linked in. |
|  | **7** | conversions of referrals, I get a lot of referrals from SMOs directly and very rarely will those patients fail to attend, and and you will always get good conversion when the referral comes from that authority. |
|  |  | Sometimes we'll get lovely referrals from the nurses on the treatment floor because they've spent 4 h with the patient, and they've had in depth discussions. And they've actually been able to take the patient from pre-contemplation to contemplation, to preparation. To then, ‘Oh, yeah. Well, okay, how about you refer me to that person’, and when they do treatment education here, there will often be like a knock on my door. If my door’s open the nurse that's been doing nivolumab or pembrolizumab or divolumab, or any of those ipilimumab educations with patients, they say, ‘Now this is [participant name]. This is the guy I spoke to you about before and he's got a gym here. But you don't have to be in the gym. He he can just talk to you, and he can set you up with some stuff to do at home. So a lot of that priming of the patient is done by my team for the reasons that they've discussed with the patient and things that have come up in in those conversations around, wanting to continue to walk at the beach or having issues working in the garden. |
|  | **8** | so we have half a physio FTE in our cancer center. But at the moment it's based off referrals. So that's really dependent on the nurses and the doctors finding the patients that I think we need to see |
|  |  | I think we spend a lot of time, I guess, in an inpatient setting, educating our nurses on the ward. But I think being able to do more education in the cancer center, with the cancer CNCs, and also the, you know, registrars and trainees that go through would be helpful to get them aware of what we do in our service and getting them to refer earlier, I think would also be helpful. |
|  |  | Not so much. I think our medical oncologists are really quite pro allied health. So they're really helpful and our nurses are also really proactive and you know, encouraging. So I think, on the whole, I think people are quite helpful and probably more of a positive influence than in a negative |
| **Telehealth access** | **6** | We can offer them an option if they don't want to come to us face to face, or if they're out of the area, we can offer them a telerehab option as well, which is great. That was one of the bonuses of Covid. We all went telerehab, and so that we've kept that going as an option for patients. |
|  |  | So mostly it's face to face. obviously, if people live out of area and it's too hard for them to come as well as have ongoing treatment and then we offer them telerehab, because it seems as if a lot of the pulmonary rehab programs in rural and even our smaller metro hospitals haven't quite got back to normal. And so and often these patients I find are further down on the list, on the waiting list. So if they want to come to us I’ll take them, because I don't want to be waiting too long. |
|  | **7** | You know, patients and their families are willing to engage in telehealth if travel and distance and appointment burden have compounded a little bit. So I'll extend my services to to phone and and telehealth services as well, and that that is all about building, you know, relationships with the patients that when they are in here. So you know some patients that live a bit further away. I'll I'll engage with them through those forms. |
|  |  | the health service will hit me with insurance things like you could only supervise 6 or 8 people at once, whereas, you know, in an E context when they're at home. And we've we've we've established that we're going to use certain equipment I’ve seen these people and done initial assessment, prescribed them exercises. I then can increase that total number of participants. |
|  | **8** | Definitely infection is a big concern, I think. Particularly post COVID for a lot of patients, I think, being able to have a telehealth option is great. And I think also specifically in [state name] ‘cause we've got lots of regional and remote patients and not always the infrastructure in their local areas to be able to, you know, do rehabilitation close to home. |
| **Adherent patients** | **5** | there's a little bit of emphasis on the patient to be motivated to come and to book in. yeah. And I think that's sort of what I and I think sometimes when sort of the patients have come sort of through to us is they're not wanting to do to that. |
|  |  | The challenge with that is, I don't feel that you necessarily particularly, maybe with those patients who need it more then, they’re not necessarily going to be in a situation where they're going to be pushing themselves a little bit if they're that way inclined |
|  | **7** | So so a proactive patient and I’ll say proactive because there needs to be a fair bit of proactivity in in prehabilitation, has access to my services in in the gym prior to their infusion. And that will require some coordination and commitment on on their behalf. |
|  |  | I like to sometimes refine or or restrict opportunities for patients to sometimes engage in rehab. It puts a bit of value on it as well. |
|  |  | So sometimes with me, saying to patients, ‘You book’, makes them value the appointment and make the appointment. They’ll go, ‘like I, I tried to call, and someone else had booked’, and that makes them see that they're not the only non-small cell or small cell lung cancer patient at the. So then the next week they book and it's the same with my program |
|  |  | And certainly when, when I'm working with someone that's complex. I want them to, to at least be compliant with the prescriptions and and the the the plan, the plan for them that has been made by all these, you know, public servants that are trying their best |
|  |  | But I would I would say that, you know, pulmonary oncology, you know, lung cancer patients, and and even to some extent, mesothelioma patients are relatively compliant. |
| **Facility and equipment** | **4** | We're in a full, fully equipped gym, which is, we're pretty lucky. Lots of space. |
|  |  | And it's, it's kind of an existing service, and that we, you know, we didn't need any more equipment. We didn't need a new gym. Everything was already there. you know, we had the admin team. |
|  |  | I think just this space, and I know some places might not have space, but also just having the space that you not crowded in one on top of the other. I think, especially with patients, if they are a bit concerned about social distancing. So that's a big thing. Sort of you know and it's not as noisy, if you've got sort of space so that you can hear each other. Yeah, they're the sort of environmental things, but nothing else. Just yeah. Access to equipment. You know, portable sats probes, things like that |
|  | **5** | Yes, we, we have a gym. We have a very well equipped gym |
|  | **6** | It's just a very, we’ve got an old gym, but a very big gym. It's a very welcoming space. |
|  |  | So it it's sort of quite a welcoming environment. |
|  | **7** | So we in 2017 built a a a facility purpose fit for oncology patients. And oncology patients, oncology patients and hematology patients only and that's to, you know, ensure access at any time. |
|  |  | And that's you know, the the gym environment, for example, is we've got 2 treadmills to recumbent exercise bikes 2 upright exercise bikes. We've got a concept 2 rowing machine, and we've got an arm ergometer, a water resistance arm ergometer. And then we've got our obviously weight machines and and free weights as well as as I mentioned before, the therapy bands and the Swiss balls, and those sorts of things that go with it as well. The formal environment that patients can train in is is very well equipped to to get it a a good amount of aerobic, cardiovascular cardioplumonary stimulation |
|  |  | the room is well equipped with equipment that patients can use that's going to be safe for them |
|  |  | We have one of those large sort of Phillips TVs on wheels, a computer, like, you probably have at universities and things like that and yeah, that that that works really well |
|  | **8** | The only thing is that our gym space is quite small. So the telehealth groups are still run within the gym, so I guess in an ideal world having a bit more space would be nice. |
| **Class times** | **7** | I mentioned before I run the group from 1030 to 1130, because they're not having to share the roads and the car parks with health care staff that are coming to work early, and they're not having to navigate through school zones. So if it's a physical group there's things that I know that will lead to its success. |
| **Treatment experiences** | **7** | Sometimes it'll it'll be nice to actually see them after they've had the experience of an infusion. |
|  |  | And that's that's particularly where I've observed in some of the you know, nivolumab, pembrolizumab, divolumab patients they start to engage in allied health services a bit more later on into their their treatment plan. When things have settled and they've gained a predictability about their week. And we'll we'll often, you know, find engagement increases |
| **Groups allow prioritisation** | **7** | There's there's no denying that a a rehab or prehab program, you can see more people at one time. So that's, that's quite good for for the business of health care. It also can reduce, as I mentioned, you know, lower level review appointments into that setting. |
|  |  | If you can see 8 patients in an hour that's almost the whole day of 1 h for 8 patients. And then you've still got a remaining 7 h to do those things. |
|  |  | with the groups that when the when the pulmonary programs or any of the oncology rehab programs are running at capacity, one therapist or one clinician can see quite a few people in a day and and and and stretch that kind of value of care. You you, you might argue in one breath that a group is low value care. And then you might also value in another breath that it's high value care because you're seeing X amount of people in one moment |

Table S4. Barriers to rehabilitation

| **Subtheme** | **ID** | **Quotes** |
| --- | --- | --- |
| **Patient Participants** | | |
| **Cost** | **3** | Look it, it costs a lot of money, you know, because I'm doing this privately the GP did give me some more sessions, but I think it's you know, it's insignificant, really [laughing]. So there's not much funding for that sort of stuff, but not that I expect it. I don't. You know, I can manage to pay it myself, but it's just a bit of a I'd probably do more if it wasn't so expensive. |
|  |  | a lot of the programs where you need private insurance to access them. Many people can't. A lot of people can't afford private insurance. |
| **Not individualised** | **3** | I mean the one I did at [name of hospital] that was fairly unhelpful, to really, because it was too general, it was for all lung disease and I didn't, actually hadn't been diagnosed with, with lung disease at that point. But there was people with lung cancer in, in the rehab group, and there was people with heart, heart problems as well. And there was. I don't think there was stroke people, but there was certainly heart and lung people. And it wasn't that useful because you’d get lectured about these nice basic things. You know, I used to be a psychologist and, and you know that. So this is so basic, it's just stupid, you know. I could stand up and do a better talk than they are doing [laughing]. But they don't know. You know they, they don't know the audience, and maybe they should know the audience. |
| **Lack of equipment** | **3** | I had the exercise physiologist come to my house, and I didn't think that was as useful because they haven't got the equipment with them really that you might need and it's a bit more limited. |
| **Education not relevant** | **3** | And most of the information is not relevant. You know for the others, and you have to sit through these endless stuff about, you know, having a rest when you're tired, or you know yourselves during the day, and all that kind of thing. Look, but that's relevant for everyone. But I just thought it was a bit ho hum, really. |
| **Lack of time** | **16_17** | ‘Look,we don't feel like we even have time’. And they they’re all just like ‘Hey, we have time and we’re working how come you don't? you know. And then I feel like saying, ‘Look at the calendar of what we're doing, and tell me whether you think’, |
|  |  | So I still work 2 days. So I can't cope with the thought of going for a walk before I work, unless I'm up at like when I used to do that, I'd be up at 5 in the morning to do it because otherwise like some people get up, get dressed, and whatever leave I prefer to shower in the morning. I don't want to rush myself to get to work. I can't cope with being stressed before I’ve even got there. So so you know, I don't want to be running in back into the house, jumping into a shower and running out the door. So so I, I would prefer unless I get up, you know, like at 5 or 5 30, and just do that walk and be back here in time to shower, and everything. I wouldn't do it in the morning. But then the reality is that the afternoon we are both too buggered, really. |
|  | **1** | really the only challenge is if we might have a few things on and I say to my husband, ‘I I've got my exercise class’. So we take a minute, and I calm down and and and then we you know, we we work around it to make it work. I guess that's really the only challenge I've had. |
|  |  | we've only got one car. So the time I'm thinking of is okay. Well, you drop me off and we had our granddaughter, and we had to get her to back to a place on time because she had another commitment. So it it was cool. We worked out. Okay, I can. I could be dropped, have all my gear organized, and and then they can keep going to to, you know to fit in with their schedule and that worked well, and then I just had a beautiful walk home because I live in [name of suburb] and I just walked home along [name of street] and it's a beautiful walk and up a bit of [name of street], and so we managed it that way and mmhh I was happy. It worked. |
| **Mental health** | **16_17** | One day I said to [participant name], you've got to stop wallowing in the fact that you know you're unwell. I know you're not used to being unwell but you've got to buck up and actually just start living life and stop being Mister misery guts constantly because it's just bringing us all down, you know. I know you're not used to being sick, but this is crazy, you know you you can walk. |
|  | **2** | I was angry in the beginning when I first got sick and felt like I wanted to blame everybody else. That’s when the doctor said look it’s happened, get over it and reach forward. So the effort, yeah I did have to put effort into, to make myself a bit, but not now. |
|  |  | And I used to think ‘leave me alone’. That was in my angry stage. That's in my you did this, because I wanted to blame everybody, you did this to me. You let me get this sick. You give me a pill to make it better so in the beginning I was um reluctant. |
| **Fatigue** | **16_17** | Okay, this afternoon we've got some time we could go for a walk. We're both so exhausted. We just want to sit down and rest. Do you know, like and and potentially start dozing on the couch rather than thinking, this is the time to walk. |
| **Travel distance** | **1** | I think for some, maybe distance is a problem |
|  | **16_17** | but why couldn't you do an exercise program just with someone on Zoom? Or if you had to go in, go in and do it, but it's a lot of driving in and back to do say half an hour, or an hour of exercise. |
| **Lack of social support** | **16_17** | UNITE16: And yeah, if the hospital said we want you to do that, or, you know, want you to get more exercise I'd probably do it without [participant name] I'd go for a walk. whereas now, at the moment if I say to [participant name] ‘Let's go for a walk’ she’d say ‘now doesn’t suits me’. UNITE17: because I’m too tired. UNITE16: Because I’m asking to do it when it suits me. I'm not. I'm not looking at the other side of the But yeah, I'd probably, you know I can. I'll walk. I'd walk the dog. |
|  | **18** | But if he hadn't been here or I didn't have someone here, I don't think I would have done it. Because it's frightening, not being able to get air. |
| **Breathlessness** | **18** | Because I was scared if I pushed myself too much I was scared that I wouldn't be able to breathe because I'd get so breathless that I would go too far, and I'd fall over and die, and that's the truth. |
|  |  | I thought of joining a gym, but it still bothers me that if I go too far. Because when you get breathless, you get really breathless and then for me at least, anyway, I panic, then if I can't get the oxygen in. |
|  |  | My first lap around my house, and it's just a normal average size house. I was breathless and panting and thinking I can't do this. Left it a couple of days. |
| **Weather** | **18** | the humidity, the heat, and the humidity affects me with my lungs |
| **Deconditioning/Reduced fitness** | **18** | 100%, just effort. thinking that I can't do this because my fitness level was just so low |
|  | **2** | in the beginning, when I was still so unwell, I think it seemed to be an effort |
| **Fear of over-exerting** | **18** | that fear of something happening, not being able to get that air in I think that plays, for me it played a huge part |
|  |  | you're so scared of that. You'll do the wrong wrong thing, or you'll make things worse |
|  |  | But and then I would get to a point where I think I would stop because I thought I'd gone too far and that would scare me, |
|  |  | that's that fright factor, that if you over exercise or you push yourself too much what's going to happen? |
| **Lack of supervision** | **1** | if you are needing more assistance, it it might be a disadvantage. |
| **Unfamliar habit** | **1** | Because I can't say the routine of exercise is something I find easy. |
| **Poor technology skills** | **1** | I'm not good with technology. |
| **Reduced self-efficacy** | **2** | in the very early time. It was still hard and I thought I’ve got to give up. But I didn’t know that I could physically, physically, sorry and mentally. But that was only in beginning. When I went to [name of organisation] and [name of organisation] I thought I can’t do this. I just can’t. |
| **Healthcare Professional participants** | | |
| **Allied health training - limited** | **4** | I have tried to, I did a webinar that the APA did with an oncologist about immunotherapy, and I must admit it, it was interesting, but I feel there's probably a gap. And I, you know, need to do this myself as well. But look into things that maybe something that's designed more for physios by physios. I found it, it's sort of a bit more science based and more about. Yeah. So and that says more about my intelligence than the presentation, but I probably didn't get as much from it as I, I had hoped for |
|  | **5** | And one of the things that you mentioned obviously, was the inspiratory muscle training. And I sort of went. Yes, actually, I need to look into that a little bit more as to who it's appropriate for who it's not appropriate for, so I think I will probably feel pretty confident. But I wouldn't say I was super confident, in terms of some of the additional sort of things that maybe we could add value with, and knowing beyond a lot of the what I would consider sort of regulars to rehab. What are some of those specific techniques that are related to this population that could benefit. So, so that is probably one of those, one of those specifics. So I think understanding the newer treatments. And on this and kind of having a really good understanding of some of those specific interventions. |
|  | **7** | There hasn't been too much from an allied health, this is a training program in immunotherapy. And if if there was, if there were a few of them, I'd probably still do them anyway. So any any program like that I would do whether it was a webinar or something. |
|  | **8** | So I think there's not a good collective group, either, that we can sort of sit and you know, share what we found in terms of our own experiences and literature as well, so I think that's, probably another area that we don't quite have. |
|  |  | I think it'd be great to be able to do like a lung cancer study day broadly, and then having immunotherapy as a portion of that because I've definitely attended a few of the nurses, lung cancer study days. And I always find there really good and again, I think we are siloed, and it would be good to be able to think about lung cancer patients across the spectrum of their care. From, you know, diagnosis, prehabilitation, whether that's immunotherapy or surgery, post op and then into the phase where we see them further down the track. Yeah, I think it'd be great to have something together to be able to you know, work people through that and build their confidence and broaden their scope a little bit. |
| **Carer actions** | **6** | Often I have to unbubble wrap the patient, you know, take the family off them, you know. They're killing them, killing them with kindness. And we have to say, it's okay for you to move and it's okay, you know, for you to do these exercises. It's not going to spread the cancer through your body. You know all those sorts of things. You know, those old wives tales that their family. It's giving the families a job, but at the same time it's holding them back. |
|  |  | Because I have had a patient recently where the wife said, ‘He's really too sick to do anything’. And I said, ‘Oh, you, look, we know that exercise is really important at this stage. Let’s just do a little bit each day and see how we go. And if you would like to go for a cup of tea that would be lovely. We will look after him, your husband for you.’ |
| **Cost** | **8** | We tried doing a video so that the nurses could just deliver the video rather than generally, we have a booklet that we will provide and then we'll give an individualized exercise program on top of having the booklet. We tried. Yeah, we tried looking at a video, but it was really difficult in terms of getting audio visual. And then the cost of it was just ridiculous. And so we we couldn't really. Yeah, get that. |
| **Fatigue** | **8** | But most particularly the the the key thing that I keep referring to subtly is fatigue is that sometimes attendance rates can can fluctuate, depending on where a patient is within their their fatigue |
| **Insufficient appointment time** | **5** | I think if we were referring all the patients we really, really wanted to they would get overloaded quite quickly. |
|  |  | it's like I was chatting to one of my colleagues the other day, and she was just like, ‘Oh, you know, I've run over’, and this is just it wasn't in cancer care it was something else, she was like, ‘I've run over. I just probably need to be a bit more organized in the day’. And I said, ‘no’, I said, ‘you've not run over because you've been disorganized. You've run over because you care’ and because you've spent that extra little bit of time, and I think that's probably where I'm a bit of my burnouts been is is because because you care, you go that extra little bit to go what's happening with them, you know, who's following them up? Have they been seen? Okay, when can I, you know, or what else can I do to get, you know, use of the care that you need |
|  |  | running groups is a bit of a you know. It's a bit of a burden, because you still got to then, you know, let's say, Hey, you get an hour to be able to do a group. You actually don't. You need a bit longer, because you need to be able to do the actual you know the charts and things afterwards. You know and noticing, you know what it is. You know, when you're doing pre-post testing and things sort of like that. So you know, I know when I used to work as part of a day rehab program at [hospital name] that was always the hardest sort of thing because you get however, many patients through over a 3 hour. Ching, ching, ching! Sort of like, you know, I'd sort of do, sort of 3, 1 h blocks back-to-back, and then you'd kind of have to sit down and do all the charts and sort of things sort of like that so. And making sure it's quality. |
|  | **7** | I think that there needs to be a a necessary amount of intimacy in the work that we do and and at that comes the cost sometimes, like I mentioned before, with sheer volume of work, that you you can find yourself running late and and apologizing and and the key to that for me, is actually acknowledging that the apologies are coming due to the the systems and the structures not being in place to support that. So ay, someone might be able to get an appointment done in 20 min. But just because you get an appointment done in 20 min shouldn’t mean that you allocate 20 min to that appointment. And and often where you you start to get the house of cards falling down is when when people aren't given enough time to do the task that's necessary. There's, there's probably a difference between what's needed and then what's necessary. |
|  |  | programs not being as successful as they can, because they're not always given enough time and in in successful, pulmonary rehab that I've done in the past people want that little bit of time afterwards. So if you're pushing hard this the physical rehab in that time then patients will still want the psychosocial, the peer support. So you've got to make sure that you've got chairs in the gym that people can sit down and talk on, or we need to set up some time after that, where they can just chat afterwards. But through through time you you will see that maybe a new appointment needs to be an hour, regardless of whether it could be done in 45, because you lost 15 minutes in the beginning due to emotional lability. Or you lost 15 minutes at the end, once rapport was established and all these questions just came out of nowhere. |
|  | **8** | But also what the patients have told me when I say, why didn't you tell your medical oncologist that you were struggling with your walking? Why didn't you tell them you more breathless? They say, well, I've got 15 min with my doctor, and we talk about my chemo or my immunotherapy. I just don't think that they have time, or that they’re interested in my physical function. |
| **Insufficient time for prehab** | **5** | But we were looking to see if we could try and get some of those patients in pre chemotherapy. But the window is, as we discussed, is very, very tight. |
|  |  | They then got to go through that paper trail. We can very easily lose 2 weeks there. So what we were sort of finding when we were doing this is, we've only got a small window before someone might start chemotherapy. That window might be 2 weeks. And the reason we wanted to try and get them, at least for the 2 weeks beforehand was is, we know that if we start someone on a prehab program the week they're starting chemo it's just too much for them there. We need to get them into that, seeing what they're capable of doing in those 2 weeks at least, beforehand. Get them into that psyche of doing a little bit of something. |
|  | **6** | I’d love to see more prehab and I think, I think that the trouble with that is often the surgeon’s see the patient and say, ‘we'll operate tomorrow’, so there's no time for prehab, |
| **Lack of managerial support** | **5** | Well, can we do a 4 week, pre-chemo possible telehealth, prehab sort of program for them? And we kind of came up with the idea. But when we tried to look at the specifics of putting it into it, this is where we hit those brick walls. So one was actually getting the buy in from more senior management to go ‘We want to run these groups’ and to allow, we wanted it to be combined across the 2 physios. So across the 0.8 that we had FTE, for I guess the 0.8 of EP. That was this at the sort of particular service, or he has one full, full time equivalent, but it's across 2 sides. Go can we share that load between 2 EP sessions and 2 physio sessions across a rotating fortnightly block. So, getting the permission to do that was a bit slow and wasn't particularly forthcoming. |
|  |  | We can kind of get this system working and not get overloaded with [inaudible] but from a I guess, our sort of director sort of thing going ‘Well, are you going to see enough patients?’ to ‘What are the activity levels?’ what the, it becomes then from an appointment and I guess activity quantifiable. Other things. The ironic thing is, if we can get it up and running, it will actually really boost our activity levels. In addition to boosting patient care. |
|  |  | So see the fine example of I'm doing this on one of my non-work days. So I guess that shows you the level of support we've had |
|  |  | So I'm essentially I'm a grade 2 physio, that might not be quite where you had your grade. 3 wants to take things. So anything you do is got to be sustainable on what they wanting to do. It feels like it's very much individual interest as opposed to going, ‘Yes, as a physiotherapy service, the oncology team are saying, we want prehab rehab services. So you need to provide them. How are we going to do it?’ |
|  |  | There is nothing from what I've experienced so far within cancer care that says, and whether that's lung or other tumor streams, saying that we have a standard pathway of care that these patients attend because they do. They do cardiac rehab you know. They go twice a week for 4 weeks. Then that is, that has got a physio attached to it that has got nursing attached to it. It has got dedicated time and resources. But there is not that within cancer, and there should be so, I think but when the Health Service itself realizes and prioritizes that, I think that it will change, I think until then we will be fighting a battle constantly. |
| **Lengthy referral processes** | **5** | We then also hit the roadblock of admin. So the way patients are referred to us at the moment, it is an electronic referral system. However, when it gets to our admin officers, who are doing the, who, who receive those. So those referrals don't come directly to us as a clinician. They get put into the admin system. They then get physically printed out. They get put in a tray which we've got to physically pick up, physically triage, physically put back in a tray, to then be inputted by the admin assistants to then be contacted, and offered an appointment which could then, at the moment my clinics are booked for the next 2 weeks solid. So you've kind of got to go from, patient's initial appointment with either oncologist and then care nurse coordinator to double check that they're going to be appropriate for referral in from a medical point of view. They then got to go through that paper trail. We can very easily lose 2 weeks there. |
|  |  | So we were finding that when we looked at what the practicalities of it we'd already lost 2 weeks before they'd even get to us. So I think our big barriers were admin, and that whole sort of streamlining that process to getting the patient in. |
|  | **6** | If they are, a lot of people are referred to us and don't want to come to us face to face, but want to go to the local pulmonary rehab, well we will refer them on fairly quickly, and we sort of always say, ‘Could you please see these patients as soon as possible?’ And I will, you know, even though we have a waiting list, I always say it. If I get these patients I always put them in very quickly, again because you know that delay often causes a lot of, a lot more problems for them |
| **Limited services** | **5** | But again, those classes are only run twice a week. at one of our, at our main center. So we have, we have 3 hospitals that provide cancer care actually, it just services. definitely 2 sorry that certainly provide at least chemotherapy. We'll have some patients who will have surgery any one of 3 hospitals, and but radiation is only available at the, at the one main hospital |
| **Lung cancer stigma** | **8** | And I do think it's a problem in that, self-worth, particularly for the patients that have been heavy smokers. Sometimes don't feel like you know, they, that deserve the rehabilitation, or that that feeling that they brought it on themselves so they should just, you know, carry on and and not do things to try and improve |
| **Poor technology skills** | **7** | the telehealth could be done more and better. It's just support around that. I I would like in the next 6 months to have more of a a telehealth oncology rehab prehab program than what I've already got, the the limitations to that are just that the the time constraints. The the telehealth that I run at the moment it's okay wen you've got one person and they've got connectivity issues. And you call them. And you say, just close it down, open up the email again. Click on the link. Your microphone is not working. When I've done groups with 19 people, and you've got 10 people that are sweet, good to go. And then you've got 2 people that they just can't hear you. And you you really need off-sider support for that. So as the therapist you can say, ‘we're going to go forward and [interviewer name] is going to call you, and and you're gonna come in and we'll probably, you'll probably miss one or 2 exercises’. |
|  |  | In the in the pulmonary rehab that I do on a Thursday morning. They have the CN, that is there because it's a nurse led program fed into by the physio. So the the physio or the EP, myself, we will run the class, run the program, and and any issue the CN will just get on to that and get the person back on so. |
|  |  | and the key to that is is the allocation of the extra FTE to to ensure the administration. |
| **Travel distance** | **5** | I guess one of the other barriers we were talking about is a geographical spread of patients. That was the only thing I was going to say that we kind of came up for, and burden of appointments of patients because of where we are on the [location name]. It's a little bit different to say, like [city name], where you've got metropolitan, I know goes all the way down to [town name], you know you're still in [city name] when you are [town name] and and sort of that sort of ability through here. We've got patients who can very easily travel, for you know, an hour and a half, 2 hours to get to an appointment. our [location name], and sort of wider [location name] patients come from all over, and we're talking of very long distances that some of them have to travel. So that was our, that was our other part with that. That's why we started to maybe look at telehealth. |
|  | **7** | the attendance to the physical gym extends to about half an hour from the hospital and the health service extends much beyond that. So it comes back to that inequity again, that the the pulmonary rehab, the the oncology rehabs, and all of those sort of rehab programs are still will attended, but they're attended by people that live close |
| **Appointment burden** | **5** | sometimes by the time they've got to us they've got a lot of appointments, and they've had a lot of overloads sort of of that. So it's trying to sort of look at some of those practicalities of how they can put that into place. |
|  |  | And the reason we wanted to try and get them, at least for the 2 weeks beforehand was is, we know that if we start someone on a prehab program the week they're starting chemo it's just too much for them there. |
|  |  | The patient’s buy in, to also then go, ‘I've got lots of appointments. If this is not a standard part of my pathway. I've got too many other things. I've got too many appointments. I've got to go for scans. I've got to go for blood tests. I've got to go for this’ |
|  | **6** | Obviously, I try and fit in around their treatment times and so that they just not too fatigued. |
|  | **7** | Obviously, they they’re chemotherapy or their immunotherapy infusion dates, so patients can get get quite overwhelmed with appointments, certainly with the breadth of our MDT. |
|  |  | the the cognitive change, the cognitive behavior change I'm seeking because of. I've got all these appointments. I've got to get my bloods taken this week. I've got you know all these other appointments. And now you want me to do all this. There's already so much behavior change being asked of that person. |
|  | **8** | We definitely have trouble already getting to people on a one-to-one basis, because they just don't want to stay in the hospital for any longer than I need to. |
| **Workload** | **5** | our other brick wall is we're not given any additional time to work on this, so that comes back to our pre-recording conversation about this is essentially when we're looking at doing this because we don't have a dedicated pathway for this. It's essentially it’s a bit of a quality project to go, we're going to take this patient cohort. We want to explore what the benefits are because it's not part of routine care at the moment. And we're looking at trying to do something that's outside of our normal routine care. Which is ridiculous. So to actually have the time to work through, to problem solve all of these potential barriers. We're having to do that outside of our clinical time. Which is already pretty loaded like, I say, you know I very rarely get out of work on time, and I'm not allocated any additional time for any quality projects, or for exploring these sides of things. |
|  |  | And the reason that I didn't push to get this to where we wanted to be, for the lung conference was I was doing this in my own time, and it hit a personal wall between work, life, family balance, and I just sort of went. You know what I've got to just, I've got to find that balance |
|  |  | I think my challenge has been that I have been in that particular role 2 days a week. I feel like I probably haven't put as much in recently because I sort of was hitting a bit of a brick wall, and I needed to focus on other things. |
|  | **7** | the workload that I currently absorb is haematology, oncology and all the tumour streams that come with that. So whilst I do as much as I can for pulmonary rehab there’s some limitations on, you know, service expansion and quality improvement projects around that. |
| **Organisational structure and factors** | **5** | I think the problem is is that Physio and EP and technically sit outside of cancer care services. So we report to our director of physiotherapy. We don't report directly at all to cancer care. We don't have a representative that sits within the cancer care directorate. So there is no one who is having these conversations with the oncology team saying What do you need from us? |
|  | **8** | But I think the hospital's goal is probably different to mine in that a lot of it is around discharge, focus |
